# A FoxA2+ long-term stem cell population is necessary for growth plate cartilage regeneration after injury

**DOI:** 10.1038/s41467-022-30247-1

**Published:** 2022-05-06

**Authors:** Shanmugam Muruganandan, Rachel Pierce, Dian Astari Teguh, Rocio Fuente Perez, Nicole Bell, Brandon Nguyen, Katherine Hohl, Brian D. Snyder, Mark W. Grinstaff, Hannah Alberico, Dori Woods, Yiwei Kong, Corneliu Sima, Sanket Bhagat, Kailing Ho, Vicki Rosen, Laura Gamer, Andreia M. Ionescu

**Affiliations:** 1 Department of Biology, 134 Mugar Life Sciences Building, Northeastern University, 360 Huntington Ave, Boston, MA 02115 USA; 2 Centre for Advanced Orthopedic Studies, Beth Israel Deaconess Medical Center, 330 Brookline Avenue, Boston, MA 02215 USA; 3 Division of Pediatrics, University of Oviedo, Oviedo, 33206 Spain; 4 New York University College of Dentistry, 345 E.24th St, New York, NY 10010 USA; 5 Moderna Therapeutics, One Upland Rd, Norwood, Ohio, MA 02062 USA; 6 Departments of Biomedical Engineering, Chemistry, and Medicine, Boston University, 590 Commonwealth Ave, SCI 518, Boston, MA 02215 USA; 7 Department of Orthopedic Surgery, Boston Children’s Hospital, 300 Longwood Ave, Boston, MA 02115 USA; 8 Department of Oral Medicine, Infection, and Immunity, Harvard School of Dental Medicine, 188 Longwood Avenue, Boston, MA 02115 USA; 9 Ultragenyx Pharmaceutical, 840 Memorial Drive, Cambridge, MA 02139 USA; 10 Department of Developmental Biology, Harvard School of Dental Medicine, 188 Longwood Avenue, Boston, MA 02115 USA

**Keywords:** Mesenchymal stem cells, Cartilage development, Regeneration

## Abstract

Longitudinal bone growth, achieved through endochondral ossification, is accomplished by a cartilaginous structure, the physis or growth plate, comprised of morphologically distinct zones related to chondrocyte function: resting, proliferating and hypertrophic zones. The resting zone is a stem cell-rich region that gives rise to the growth plate, and exhibits regenerative capabilities in response to injury. We discovered a FoxA2+group of long-term skeletal stem cells, situated at the top of resting zone, adjacent the secondary ossification center, distinct from the previously characterized PTHrP+ stem cells. Compared to PTHrP+ cells, FoxA2+ cells exhibit higher clonogenicity and longevity. FoxA2+ cells exhibit dual osteo-chondro-progenitor activity during early postnatal development (P0-P28) and chondrogenic potential beyond P28. When the growth plate is injured, FoxA2+ cells expand in response to trauma, and produce physeal cartilage for growth plate tissue regeneration.

## Abstract (web summary)

The growth plate cartilage supports long bone growth. Here the authors identify FoxA2+ long term stem cells in the growth plate that are stratified with short term PTHrP+ cells, participate in production of hyaline cartilage, expand in response to trauma, and whose ablation impairs cartilage regeneration.

## Introduction

Longitudinal bone growth is achieved by endochondral ossification, implemented by the physis or growth plate (GP), a cartilaginous structure located between the epiphysis and metaphysis at the ends of long bones forming the appendicular skeleton. Human GP is comprised of five morphologically distinct zones: resting (RZ), proliferation (PZ), hypertrophy (HZ), calcification (CZ), and ossification (OZ)^1^. Physeal injuries are a major cause of skeletal morbidity in growing children, manifesting as angular or shortened limb deformities, due to partial or complete arrest of GP function^2^. Clinically, GP injuries are classified according to the Salter–Harris (SH) system, predicated on fracture morphology involving the physis ± epiphysis and/or metaphysis: SH1) physeal shear separation; SH2) physis + metaphysis; SH3) physis + epiphysis; SH 4) physis + epiphysis + metaphysis; SH 5) crush injury to physis. SH1 fractures have the best prognosis, healing without disrupted growth. The cleavage plane is primarily through the HZ, structurally the weakest zone comprising the GP, owing to lack of calcification and limited collagenous matrix. Stiffened by a collagen matrix, the adjacent RZ and PZ are unaffected^3,4^. In contrast, SH 3–5 injuries disturb all zones, resulting in disturbed bone growth^3,4^. These clinical scenarios suggest that involvement of the RZ affects the ability of the GP to regenerate in response to injury. In rabbits, even when the PZ and HZ from ulnar GP were excised, the restoration of the GP was still possible, as long as the RZ remained intact^5,6^. As the name implies, the “resting zone” houses a quiescent progenitor cell population^1^. Several studies have identified in mouse^7^, rat^8^, and rabbit^9^ slow-cycling cells present in the RZ immediately adjacent to the secondary ossification center (SOC). In pulse-chase experiments, animals were administered [^3^H] thymidine or 5-bromo-2-deoxyuridine (BrdU) for a prolonged period sufficient to allow slow-cycling stem cells to complete one cycle. At the end of the chase, the label diminishes in proliferating cells, but remains undiluted in slow-cycling cells. While these reports demonstrate the presence of slow-cycling, potential progenitor cells at the RZ, their genetic signature remains undefined, as there is no biomarker to isolate and characterize these cells.

Recently Newton et al., using clonal genetic tracing with a *col2*^*CRE;ERT;*^*R26-Confetti* mouse line and functional perturbations, established that formation of the SOC triggered GP chondroprogenitors to undergo a radical shift in clonality and to acquire self-renewal capabilities^10^. However, since “Confetti” labeling is based on collagen type II expression, an uber-marker for all GP cells, there was no unique biomarker to isolate and/or characterize individual stem cell subgroups. Next, Mizuhashi et al. employed a *PTHrP*^*Cre.ERT*^*;Tomato*^*f/+*^ mouse strain to reveal that PTHrP marked a subset of stem cells located at the bottom of the RZ^11^. Labeling, tracing and isolating RZ cells based on their PTHrP expression both in vivo and in vitro, demonstrated that a subgroup PTHrP+(positive) stem cell evolved predominantly into columnar chondrocytes^11^. Together with another yet unidentified PTHrP−(negative) subgroup located at the top of the RZ, adjacent to the SOC, PTHrP+ cells, located primarily at the bottom of the RZ, contributed to long-term GP growth^11^. In the present work, we prove that a population of FoxA2+col10−cells, located at the top of the RZ, are the PTHrP-(negative) stem cell population, and represent a subgroup of long-term skeletal stem cells (LTSSC) capable of dual osteo-chondro-progenitor activity during early postnatal development (P0-P28) and chondrogenic potential beyond P28, fundamental to GP turnover and regeneration following injury.

## Results

### FoxA2 expression prefigures SOC formation

**Fig. 1:**
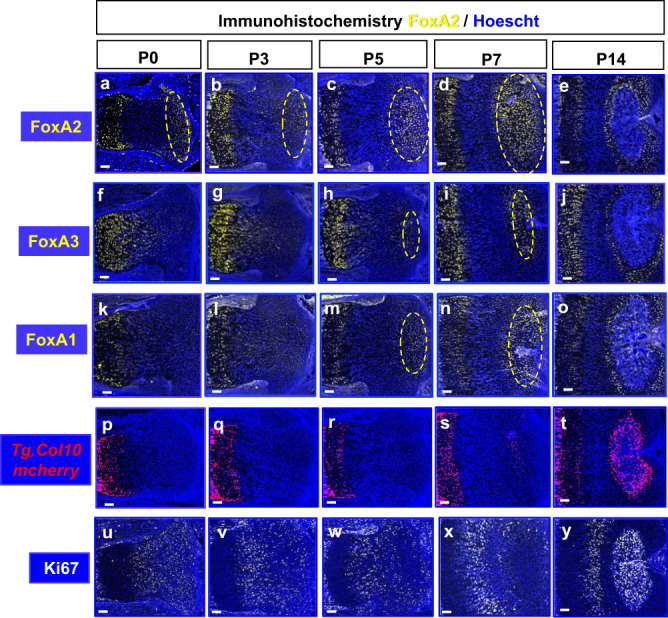
FoxA1-3 expression domain predicts the formation of the SOC. Immunohistochemistry for FoxA2 (**a**–**e**), FoxA3 (**f**–**j**), FoxA1 (**k**–**o**), Ki67 (**u**–**y**), and fluorescence microscopy on *Tg.col10a1*^*mcherry*^ mice (**p**–**t**) on postnatal day P0, P3, P5, P7, P14 tibia sections. FoxA1-3 (yellow), Ki67 (white), Hoechst (blue), mcherry fluorescence (red). Scale bars, 100 µm.

We previously discovered that FoxA transcription factors are key regulators of chondrocyte hypertrophy^12^. FoxA1-3 are highly expressed in the hypertrophic zone (HZ) of newborn mice GP (Fig. 1a, f, k). However, unlike FoxA3, which is expressed in a broader domain throughout the GP (Fig. 1f), or FoxA1, which is highly expressed in the HZ but very little elsewhere (Fig. 1k), FoxA2 is expressed in two separate domains: the HZ and a discrete periarticular domain located at the ends of the long bones (Fig. 1a). Throughout postnatal development (P0-P14), this distinct FoxA2+population is continuously enlarging, possibly prefiguring the formation of the SOC (Fig. 1a–e). FoxA1 and FoxA3 also correlate with SOC development, but emerge in the epiphyseal cartilage around P5 (Fig. 1f–o), much later than FoxA2, which is expressed from birth (P0) (Fig. 1a–e). In the presumptive SOC, the first col10+ cells appear by P7, as shown in *Tg.col10*^*mCherry*^ mice, but FoxA2+ cells appear as early as P0 (Fig. 1p–t). Comparison between FoxA2+ cells in the periarticular region with the columnar GP cells, did not reveal any histological or morphological differences and no noticeable change in Ki67 expression and proliferation, prior to P5 (Fig. 1u–y). However, after P5 FoxA2+ cells downregulate the expression of Ki67, in preparation for hypertrophic differentiation (Fig. 1w, x). Altogether, these experiments suggest that FoxA2 expression marks the epiphyseal cartilage, most of which will form the secondary ossification center, far sooner than col.10 expression.

### FoxA2+ cells give rise to the GP RZ top compartment

**Fig. 2:**
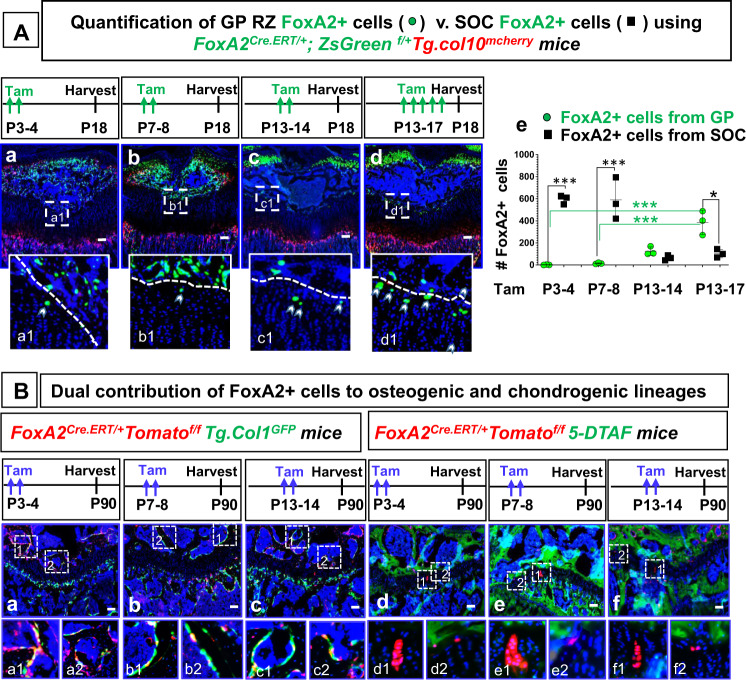
A population of FoxA2+col10− cells prefigures SOC formation and gives rise to the top compartment of the GP RZ. **A** Tibia sections harvested from *FoxA2*^*CreERT2/+*^*;ZsGreen*^*fl/+*^*; Tg.Col10*^*mcherry*^ mice treated with 2x tamoxifen (Tam) injections at postnatal days P3-4 (**a**), P7-8 (**b**), P13-14 (**c**), or 5x tamoxifen injections P13-P17(**d**), and harvested at P18. Hoechst (blue), ZsGreen fluorescence microscopy (green), mcherry fluorescence microscopy (red). Scale bars, 100 µm. Representative details, from the interface between GP and SOC, are shown in numbered insets (**a1**–**d1**). Quantification of the number of FoxA2+(ZsGreen+) cells in GP is achieved by counting ZsGreen+ cells located in GP (extending 100 µm away from the GP/SOC interface, towards the GP). The number of ZsGreen+ cells represents a sum of *n* = 8 sections per mouse hindlimb. Similar quantification is performed for FoxA2+ cells in SOC. Data presented as mean ± SD, *n* = 3 mice. The asterisks indicate significant difference: One-way ANOVA, Tukey test; ***p  =  0.000003 (P3-4 GP vs P3-4 SOC), 0.000005 (P7-8 GP vs P7-8 SOC), 0.000569 (P3-4 GP vs P13-17 GP), 0.000828 (P7-8 GP vs P13-17 GP), and *p  =  0.011031(P13-17 GP vs P13-17 SOC). Complete statistical information is provided in Supplementary Table 1 (**e**). **B** Tibia sections from *FoxA2*^*CreERT2/+*^*;Tomato*^*fl/+*^*;Tg.Col1*^*GFP*^ mice treated with 2x tamoxifen injections P3-P4 (**a**), P7-P8 (**b**), P13-P14 (**c**), and harvested at P90. Tibia sections from *FoxA2*^*CreERT2/+*^*;Tomato*^*fl/+*^ mice treated with 2x tamoxifen injections P3-P4 (**d**), P7-P8 (**e**), P13-P14 (**f**), harvested at P90. Tomato (red), Hoechst (blue), GFP (green) (**a**–**c**), 5-DTAF (green) (**d**–**f**). Scale bars, 100 µm. Representative details are shown in numbered insets (**a1**–**f1**).

To determine whether FoxA2 marks the fate of the cells destined to become SOC, we labeled and traced FoxA2+ cells, using a *FoxA2*^*CreERT/+*^mouse line consisting of a tamoxifen-inducible CreERT2 driver knocked-in the FoxA2 3’UTR^13^. Two tamoxifen pulses, administered P3-4 or P7-8, to *FoxA2*^*CreERT/+*^*;ZsGreen*^*fl/+*^*;Tg.col10*^*mcherry*^ mice gave rise, by P18, to ZsGreen+ cells present primarily in the SOC, with a few ZsGreen+ cells present at the top of the GP RZ (Fig. 2A(a, b, e)). It was previously published that hypertrophic chondrocytes become bone cells and contribute to the osteogenic lineage^14–16^. FoxA2 is not expressed in the epiphyseal bone of the SOC, but instead it is restricted to the GP hypertrophic cartilage and a small discrete domain at the end of the long bones, as shown by immunohistochemistry for FoxA2 (Fig. 1a–e). Thus, the presence of FoxA2+progeny in the epiphyseal SOC it is likely the result of FoxA2+ hypertrophic chondrocytes contributing to the osteogenic lineage. To confirm this, we administered 2x tamoxifen injections to *FoxA2*^*Cre.ERT/+*^*;Tomato*^*f/f*^*;Tg.col1*^*GFP*^ mice at P3-4, P7-8, or P13-14 and we followed them 3 months later, demonstrating that Tomato+progeny (derived from FoxA2+ cells) are col.1+expressing bone cells (Fig. 2B(a–c)).

By P13, during SOC enlargement, we see a significant build-up of ZsGreen+ cells in the GP cartilage. In *FoxA2*^*CreERT2/+*^*;ZsGreen*^*fl/+*^*;Tg.col10*^*mcherry*^ mice injected with tamoxifen P13-14 and harvested at P18, we counted 126 ± 40 cells/hindlimb. Five tamoxifen injections, administered P13-17 to *FoxA2*^*CreERT2/+*^*;ZsGreen*^*fl/+*^*; Tg.col10*^*mcherry*^ mice, led to a further increase in ZsGreen+ cells (387 ± 109 cells/hindllimb) (Fig. 2A(c, d, e)).

As FoxA2+ cells are located on the border between GP and SOC, we sought to delineate the cartilage/bone interface by counterstaining with 5-DTAF(4,6-Dichlorotriazinyl Aminofluorescein) dye, which labels efficiently the bone matrix but not the cartilage matrix in *Aggrecan*^*CreERT/+*^*;Tomato*^*fl/+*^ mice (Supplementary Fig. 1A(a)). A P13-17 tamoxifen pulse administered to *FoxA2*^*CreERT2/+*^*;Tomato*^*fl/+*^ mice, revealed that 75% of FoxA2+ cells are in the GP cartilage, outside the 5-DTAF labeled bone domain, and 25% are in the green labeled SOC (Supplementary Fig. 1A(b, c)). This FoxA2+ population located at the top of the GP RZ, at the cartilage/bone interface, it is not only present in mice, but in rabbits as well (Supplementary Fig. 1B).

To investigate whether the FoxA2+ cells present in the RZ in early postnatal development (P0-P14) still persist in the GP cartilage 3 months later, we performed a long-term pulse chase of the *FoxA2*^*CreERT/+*^*;Tomato*^*fl/+*^ mice. Tamoxifen pulses at P3-4 (during SOC prefiguration), at P7-8 (SOC hypertrophy), and P13-14 (SOC enlargement) to *FoxA2*^*CreERT/+*^*;Tomato*^*fl/+*^ mice gave rise to Tomato+ labeled cells which, 3 months later, were still present in the RZ either as single cells, clusters or columns of progeny (Fig. 2B(d–f)), or became col.1+ bone cells in the SOC (Fig. 2B(a–c)). Altogether, these experiments suggest that a FoxA2+ population, left behind from epiphyseal cartilage postnatal development (during SOC prefiguration, hypertrophy and enlargement), contributes to the GP RZ top compartment.

### FoxA2+ long-term stem cells v. PTHrP+ short-term stem cells

**Fig. 3:**
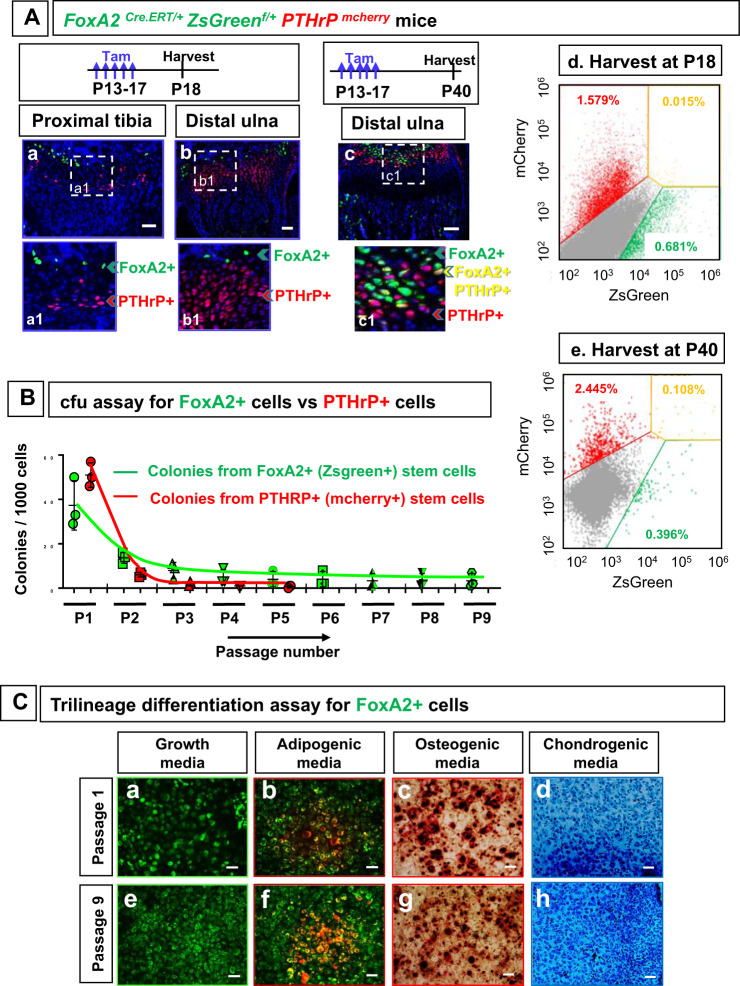
FoxA2+ cells are long-term stem cells, and they are distinct from the short-term PTHrP+ stem cells. **A** Tibia (**a**) and ulna (**b**) from *FoxA2*^*CreERT2/+*^*;ZsGreen*^*fl/+*^*;PTHrP*^*mcherry*^ mice treated with tamoxifen (Tam) P13-P17 and harvested at P18, and from *FoxA2*^*CreERT2/+*^*;ZsGreen*^*fl/+*^*;PTHrP*^*mcherry*^ mice treated with tamoxifen P13-P17 and harvested at P40 (**c**). Florescence microscopy for ZsGreen (green), mcherry (red), Hoechst (blue). Scale bars, 50 µm. Separation of FoxA2+(green) cells from PTHrP+(red) cells and double positive FoxA2+PTHrP+(yellow) cells by Fluorescence-activated cell sorting (FACS) of GP cells isolated from *FoxA2*^*CreERT2/+*^*;ZsGreen*^*fl/+*^*; PTHrP*^*mcherry*^ mice, injected with tamoxifen from P13 to P17, and harvested at P18 (**d**) or P40 (**e**). **B** Colony forming unit (cfu) assay for FoxA2+(ZsGreen+) cells (from *FoxA2*^*CreERT2/+*^*;ZsGreen*^*fl/+*^*;col.10*^*mcherry*^ mice treated with tamoxifen P13-P17 and harvested at P18) and PTHrP+(mcherry+)cells (from PTHrP^mcherry^ mice harvested at P18). Quantification of the number of colonies (per 10^3^ cells) presented as mean ± SD from *n* = 3 experiments. **C** Trilineage differentiation of FoxA2+(ZsGreen+) clones, from passage P1 (**a**–**d**) and passage P9 (**e**–**h**). Four independent clones, for each passage, were tested in either growth media (**a**, **e**), or adipogenic (**b**, **f**), osteogenic (**c**, **g**) and chondrogenic (**d**, **h**) media. Scale bars, 100 µm.

Given that FoxA2+ cells reside in the RZ, a known location for stem cells^5,11^, and that FoxA2+ cells are located in close proximity to the SOC, which is viewed as a signaling center of RZ stemness^10^, we asked whether FoxA2+ cells are different than the previously characterized PTHrP+ stem cell subset^11^. Using a *PTHrP*^*mcherry*^ mouse line, Mizuhashi et al. have recently shown that PTHrP+ cells are restricted to the bottom of the RZ, and they are dedicated, at least to some degree, to giving rise to columns of progeny^11^. Here we show that FoxA2+ cells are located at the top of the RZ and they are geographically separated from the previously characterized PTHrP+ cells^11^, in both tibia and ulna of *FoxA2*^*CreERT/+*^*;ZsGreen*^*fl/+*^*;PTHrP*^*mcherry*^ mice (Fig. 3A(a, b)). To confirm our histological findings, we performed FACS analysis on cells isolated from GP tissue of *FoxA2*^*CreERT/+*^*;ZsGreen*^*fl/+*^*;PTHrP*^*mcherry*^ mice injected with tamoxifen P13 to P17, and sacrificed at P18. We sorted the FoxA2+(ZsGreen+) cells, the PTHrP+(mCherry+) cells and the double positive (DP) cells. Consistent with the histological data, we observed no overlap between FoxA2+ cells and PTHrP+ cells (Fig. 3A(d)). The percentage of double positive (DP) cells (0.017 ± 0.004%) is the same as intrinsic (background) fluorescence of double negative (DN) control cells (0.014%).

To investigate whether FoxA2+ cells can become PTHrP+ cells over time, we pulse-chased *FoxA2*^*CreERT/+*^*;ZsGreen*^*fl/+*^*;PTHrP*^*mcherry*^ mice, by injecting them with tamoxifen P13 to P17, and sacrificing them at P40 (Fig. 3A(c, e)). Histological data reveals the presence of yellow FoxA2+PTHrP+ cells, while FACS analysis confirms an 0.1% of double positive (DP) cells (Fig. 3A(c, e)), confirming that FoxA2+ cells can become PTHrP+ cells over time.

To inquire about FoxA2+ cells self-renewability and multipotency, we used triple transgenic mice *FoxA2*^*CreERT2/+*^*;ZsGreen*^*fl/+*^*;Tg.col10*^*mCherry*^, treated with tamoxifen P13-P17 and harvested at P18, that allowed either green labeling of RZ FoxA2+col10−cells, or yellow labeling of HZ FoxA2+col10+ cells (Supplementary Fig. 2A). To compare FoxA2+ cells with PTHrP+ cells, we isolated PTHrP+ cells from P18 GP tissue of *PTHrP*^*mcherry*^ mice. Both FoxA2+ cells and PTHrP+ cells isolated from GP cartilage have high levels of cartilage specific genes (*collagen type 2*, *aggrecan*) and low levels of bone markers (*collagen type 1, alkaline phosphatase)* when compared with bone marrow mesenchymal cells (BMSC) (Supplementary Fig. 2B).

In a cfu (colony forming unit) assay, RZ FoxA2+col.10-(green) cells formed distinct colonies (Supplementary Fig. 3a–c), while HZ FoxA2+col.10+(yellow) cells failed (Supplementary Fig. 3d–f). This indicates that FoxA2+ cells, from RZ not HZ, have the capacity to form clones when cultured ex vivo. Next we compared the self-renewability of FoxA2+ cells (isolated from *FoxA2*^*CreERT2/+*^*;ZsGreen*^*fl/+*^*;Tg.col10*^*mCherry*^ mice), with that of PTHrP+ cells (isolated from *PTHrP*^*mcherry*^ mice) (Fig. 3B, Supplementary Fig. 4A). At plating, PTHrP+ cells gave rise to 40% more colonies than FoxA2+ cells (51 vs. 37), but only 11% (6/51) PTHrP+ primary colonies could form secondary colonies, whereas 38% (14/37) FoxA2+ primary colonies could be further passaged (Fig. 3B, Supplementary Fig. 4B). FoxA2+colonies have higher clonogenicity and longevity than colonies established from PTHrP+ cells. About 9% (10/112) FoxA2+ colonies reach Passage 9 and beyond, whereas only 1.4% (2/143) PTHrP+ colonies could reach Passage 5 (Fig. 3B, Supplementary Fig. 4B). To inquire whether FoxA2+ clones are multipotent, individual FoxA2+ clones, from Passage 1 and 9, were further expanded in vitro, and generated Alcian-blue matrix, Alizarin red mineralized matrix and Oil Red droplets during culture in chondrogenic, osteogenic, or adipogenic differentiation media (4/4 clones) (Fig. 3C). Altogether, these experiments demonstrate that, in vitro, FoxA2+ cells have multipotency and higher self-renewability and longevity than PTHrP+ cells.

### Dual osteo-chondro-potential for FoxA2+ cells prior to P28

**Fig. 4:**
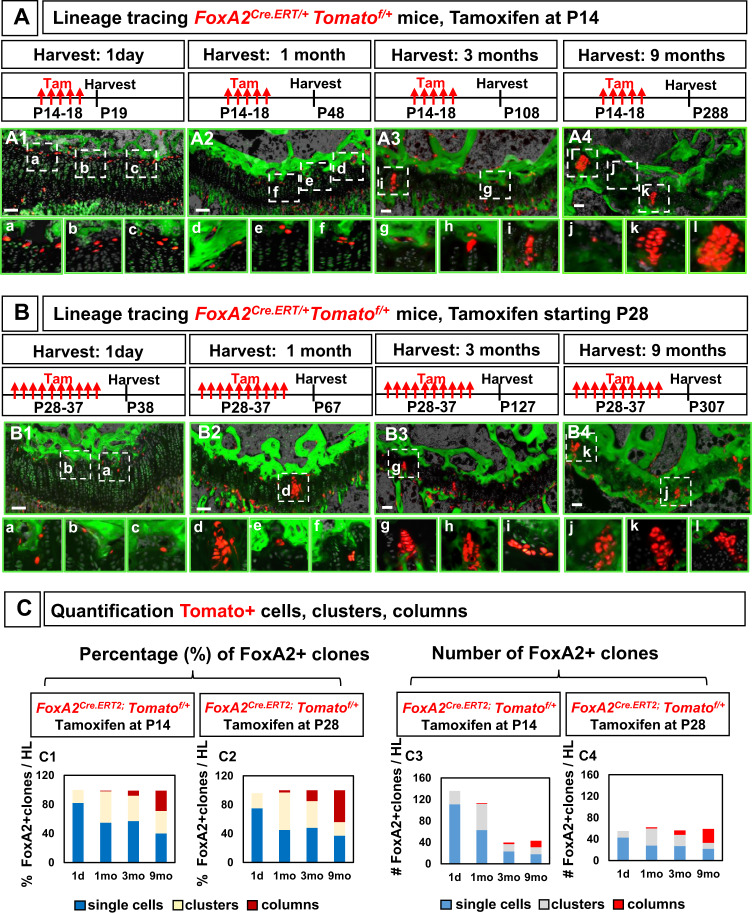
Dual osteo-chondro-potential for FoxA2+ cells in the early weeks of postnatal development (P14) and stronger chondrogenic potential beyond P28. **A** Tibia sections harvested from FoxA2^CreERT2/+^;Tomato^fl/+^ mice treated with 5x tamoxifen (Tam) injections starting at P14, and harvested at 1 day (**A1**), 1 month (**A2**), 3 months (**A3**), and 9 months (**A4**) after the last injection. Hoechst (gray), 5-DTAF (green). Bars, 100 µm. Representative details from GP sections, in numbered insets (**a**–**l**). **B** Tibia sections harvested from FoxA2^CreERT2/+^;Tomato^fl/+^ mice treated with 10x tamoxifen injections starting at P28, and harvested at 1 day (**B1**), 1 month (**B2**), 3 months (**B3**), and 9 months (**B4**) after the last injection. Hoechst (gray), 5-DTAF (green). Bars, 100 µm. Representative details from GP sections, in numbered insets (**a**–**l**). **C** Quantification of the number (**C3**, **C4**) and the percentage (**C1**, **C2**) of single cells, clusters, columns formed at 1 day, and 1, 3, 9 months after the last injection. Percentage (%) single cells is calculated by the number of single Tomato+ cells in the GP (outside the green 5-DTAF domain) to the total number of Tomato+ units (single cells, clusters and columns). Each point represents the sum of *n* = 8 sections per mouse hindlimb. Similar quantification of percentage (%) clusters and columns. One Tomato+ unit, is defined by 1 single cell, or 1 cluster comprised of two or more cells grouped together, or by 1 column of cells.

To investigate the dynamics of FoxA2+ cells clonality in vivo, we aimed to label sufficient FoxA2+ cells to demonstrate significant contribution to the GP tissue, but sparse enough to allow assessment of single clones (cells, clusters and columns of progeny) over time. Two tamoxifen injections, administered P14-P15 to *FoxA2*^*CreERT/+*^*;Tomato*^*fl/+*^ mice, labeled 7.92 ± 0.9 FoxA2+ cells, which is 8% from the total number of FoxA2+ cells detected by immunohistochemistry at P14 (97.67 ± 9 cells) (Supplementary Fig. 5A(a, b, e)). Five tamoxifen injections (administered P14-P18 to *FoxA2*^*CreERT/+*^*;Tomato*^*fl/+*^ mice) labeled 22.6 ± 4 FoxA2+ cells, increasing the labeling efficiency relative to the number of FoxA2+ cells identified via immunohistochemistry, from 8 to 23% (Supplementary Fig. 5A(a, c, e)). Five tamoxifen injections (administered P14-P18 to *FoxA2*^*CreERT2/+*^*;Tomato*^*flfl*^ mice), labeled 36.9 ± 2.3 FoxA2+ cells, increasing the efficiency of labeling from 23% (one Tomato floxed allele) to 38% (two Tomato floxed alleles) (Supplementary Fig. 5A(a, d, e)). This increase in labeling efficiency was reflected in a 2-fold increase in the number of columns in *FoxA2*^*CreERT/+*^*;Tomato*^*flfl*^ mice as compared with *FoxA2*^*CreERT/+*^*;Tomato*^*fl/+*^ mice harvested 9 months after the last tamoxifen injection (Supplementary Fig. 5B). Overall, increased labeling of the FoxA2+ cells demonstrated increased contribution of the FoxA2+ cells to the GP cartilage, but it also increased density of the Tomato+ cells hindering single clone analysis. As such, in lineage tracing experiments, we labeled a moderate amount of FoxA2+ cells by giving 5x tamoxifen injections (P14 to P18) to *FoxA2*^*CreERT/+*^*;Tomato*^*fl/+*^ mice (with one Tomato floxed allele) (Fig. 4A).

When *FoxA2*^*CreERT2/+*^*;Tomato*^*fl/+*^ mice were pulsed with tamoxifen P14-P18 and chased for 1 day or 1, 3, 9 months we observed increasing numbers of doublets, clusters, and columns of progeny (Fig. 4A). Although the initial percentage of columns is 1% at 1 month, there was substantial increase in column formation over time, and by 9 months, the columns of progeny account for 26% of the total combined structures (single cells, clusters, and columns) (Fig. 4C1, Supplementary Fig. 6C). This is in contrast with PTHrP+ cells, which gave rise to a large number of columns initially, but the number decreased significantly over time^11^. FoxA2+ cells behave as LTSSC, first giving rise to progeny, which then produce columns of chondrocytes. Consequently, the percentage of FoxA2+ clusters peaks early (43% by 1 month), but drops to 31% by 9 months, when FoxA2+ column formation is high (Fig. 4C1). Lastly, despite forming clusters and columns, a significant number of FoxA2+ cells remains as single cells. The percentage of single cells decreases rapidly over time, from 82% (1 day), to 55% (1 month), but there is still a high fraction of single cells as late as 9 months after labeling (Fig. 4C1). Altogether, these findings suggest that FoxA2+ cells constitute a more quiescent, LTSSC population located near the SOC, which gives rise to an increasing number of columns of progeny over time.

Next, we compared the clonality of FoxA2+ cells, labeled during SOC enlargement (P14-P18), with the clonality of FoxA2+ cells, labeled during SOC osteogenic maturation (P28-P37). Quantification of the percentage of FoxA2+ clones (single cells, clusters or columns) to the total number of Tomato+units, shows a similar dynamic. Both FoxA2+ cells labeled at a younger age (tamoxifen P14-18), or at an older age (tamoxifen P28-37), exhibit the same LTSSC characteristics in vivo, as shown by an increased percentage of FoxA2+ columns over time (Fig. 4A–C). However, quantification of the number of FoxA2+ clones highlights higher heterogeneity in the mice labeled at a younger age. A high number (138 FoxA2+ clones/hindlimb) are initially labeled in *FoxA2*^*CreERT/+*^*;Tomato*^*fl/+*^ mice (treated with tamoxifen P14-P18), but this number decreases significantly (to 41 FoxA2+ clones/hindlimb) by 3 months after the initial labeling (Fig. 4(C3), Supplementary Fig. 6C). In contrast, in *FoxA2*^*CreERT/+*^*;Tomato*^*fl/+*^ mice (treated with tamoxifen P28-P37) the number of FoxA2+ clones is not significantly changed between 1-day (55 FoxA2+clones/hindlimb) and 3 months (57 FoxA2+ clones/hindlimb) after the initial labeling (Fig. 4C4, Supplementary Fig. 6C). These results raise the possibility that FoxA2+ cells labeled after P28 remain mostly in GP, and their contribution to the SOC slows down significantly over time.

Indeed, for *FoxA2*^*CreERT2/+*^*;Tomato*^*fl/+*^ mice treated with tamoxifen P14 –P18, and harvested at P19, a 1-day chase reveals that 137 cells of the FoxA2+ cells are in the GP and 43 in the SOC (Supplementary Fig. 6A1). A 1-month chase increases the SOC population, from 43 to 253, suggesting that FoxA2+ cells can contribute to the epiphyseal bone, during SOC osteogenic maturation (Supplementary Fig. 6A1). This contribution is no longer endochondral, as RZ FoxA2+ cells no longer express col.10 by P21 (Supplementary Fig. 6B). Instead, FoxA2+ cells are osteo-progenitors that support directly the osteogenic lineage. This is consistent with our previous findings demonstrating that FoxA2+ cells labeled during early postnatal development gave rise to col.1+progeny in the SOC (Fig. 2B). In addition, as previously shown, the FoxA2+ cells labeled P14 to P18 give rise to clusters and increasing columns of progeny over time, suggesting chondro-progenitor quality (Fig. 4A). Altogether, these findings highlight dual osteo-chondro-progenitor fate for FoxA2+ cells labeled prior to P28.

In contrast, FoxA2+ cells labeled after P28 remain mostly in GP, and their contribution to the SOC slows down (Supplementary Fig. 6A2). For older mice, 1-day chase reveals that 55 FoxA2+ cells are in the GP and 10 FoxA2+ cells in the SOC above (Supplementary Fig. 6A2). A 1-month chase shows a similar number of FoxA2+ cells (23) in the SOC, suggesting that FoxA2+ cells contribution to the epiphyseal bone slows down significantly after P28 (Supplementary Fig. 6A2).

### Self-renewability of FoxA2+ cells in a reconstitution assay

**Fig. 5:**
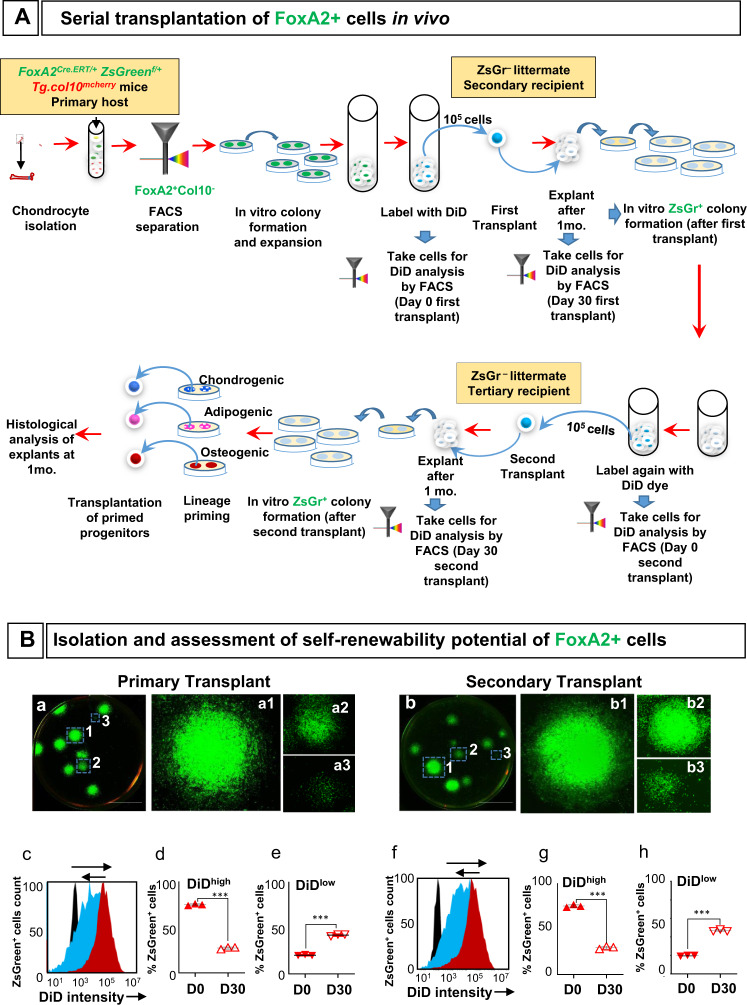
Serial transplantation of FoxA2+ cells in vivo. **A** Experimental strategy for the isolation and assessment of self-renewability and differentiation potential of FoxA2+ cells in vivo. **B** Representative images of colonies derived from FoxA2+ cells isolated from primary (**a**) and secondary transplants (**b**). Scale bars, 1 cm. DiD fluorescence intensity histogram profiles at Day 0 (red), Day 30 (blue), and negative control (black) from primary (**c**) and secondary (**f**) transplants. Percentage of DiD^high^ cells in the FoxA2+ cells from primary (**d**) and secondary (**g**) transplants. Percentage of DiD^low^ cells in the FoxA2+ cells from primary (**e**) and secondary (**h**) transplants. Data are presented as mean ± SD for each group, *n* = 3 mice. The asterisks indicate significant difference: two-tailed Student’s unpaired samples *t* test; ***p  =  7.0148E−07 (DiD^high^ primary transplant D0 vs D30), 7.84512E−06 (DiD^low^ primary transplant D0 vs D30), 3.32176E−06 (DiD^high^ secondary transplant D0 vs D30), and 3.46695E−06 (DiD^low^ secondary transplant D0 vs D30). Complete statistical information is provided in Supplementary Table 1.

To demonstrate self-renewal and multipotent capabilities of FoxA2+ cells in vivo, we performed a serial transplantation assay. We isolated FoxA2+(ZsGreen+) cells from *FoxA2*^*CreERT2/+*^*;ZsGreen*^*fl/+*^*;Tg.col10*^*mCherry*^ mice, as previously described, labeled them with DiD fluorescent dye, and transplanted them subcutaneously into ZsGreen- littermates (Fig. 5A, Supplementary Fig. 7A). After 1 month, FoxA2+(ZsGreen+) cells from the grafts exhibited self-renewability and retained colony-forming ability (Fig. 5B(a)). In addition, the grafted cells have undergone multiple rounds of cell division shown by a log-fold reduced fluorescence, observed as a shift in the intensity histogram plots (Fig. 5B(c)). Peak DiD intensity at day 0 (D0), immediately before transplantation, was 1.2 × 10^4^, and it was reduced to 1.1 × 10^3^ on day 30 (D30) post-transplantion (Fig. 5B(c)). The percentage of ZsGreen+ cells with high intensity DiD fluorescence (DiD^high^) was 75 ± 1% prior to transplantation (D0), but it was reduced to 27.3 ± 1.1% in the cells derived from grafts at D30 post-transplantation (Fig. 5B(d)). Simultaneously, the percentage of cells exhibiting low intensity DiD fluorescence (DiD^low^) was significantly increased from 20.3 ± 0.6% at D0 to 42.3 ± 1.1% at D30 (Fig. 5B(e)). This suggest that FoxA2+(ZsGreen+) cells proliferate in the primary transplants and dilute the DiD fluorescent dye.

Next, FoxA2+(ZsGreen+)cells from the primary graft were stained with DiD dye and transplanted into the tertiary recipient mice (Fig. 5A, Supplementary Fig. 7A). A similar reduction in DiD^high^ cell population (from 75.3 ± 1.5% on D0 to 29.7 ± 1.5% on D30) and a proportionate increase in DiD^low^ cell population (from 19.7 ± 0.6% on D0 to 46.7 ± 1.1% on D30) were consistent with the properties observed with cells derived from primary grafts (Fig. 5B(f, g, h)). In addition, FoxA2+(ZsGreen+) cells from the secondary grafts retain colony forming ability (Fig. 5B(b)).

The cells collected at the end of serial transplantation exhibited multi-lineage differentiation capabilities, as indicated by positive Alcian blue, LipidTox or Alizarin Red-S staining in cross-sections of tertiary grafts (Supplementary Fig. 7B(a–c)). These tertiary grafts were produced by transplantation of lineage-primed (chondrogenic, adipogenic, osteogenic) second graft-derived FoxA2+(ZsGreen+) cells (Fig. 5A, Supplementary Fig. 7B(a–c)). In contrast, second graft-derived FoxA2+(ZsGreen+) cells, without lineage priming, produced tertiary grafts that were negative for cell differentiation (Fig. 5A, Supplementary Fig. 7B(d–f)). Altogether, these experiments indicate that FoxA2+ cells preserve both their self-renewing ability and their multipotency during in vivo serial transplantation.

### GP cartilage regeneration after SH1-like injury

**Fig. 6:**
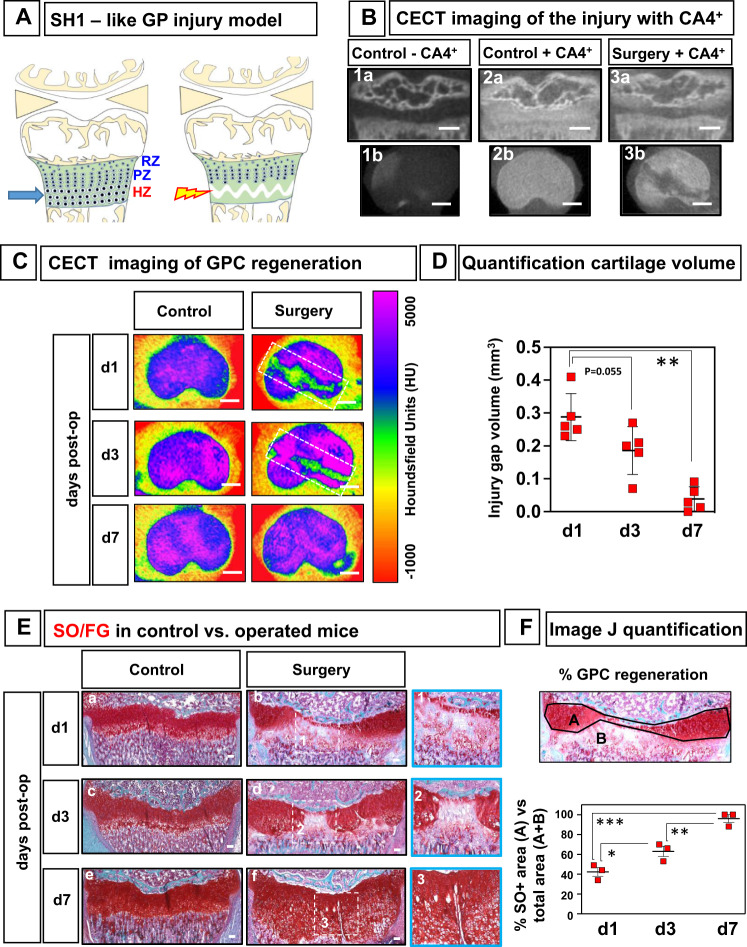
A murine model of GP injury. **A** Schematics of Salter–Harris type 1 (SH1)-like injury model. **B** CECT (contrast-enhanced computed tomography) imaging of the SH1-like injury, 1-day after surgery. Representative sagittal (**1a**, **2a**, **3a**) and transverse (**1b**, **2b**, **3b**) μCT slices of the proximal tibia, at 1-day post-surgery, before (**1a**, **1b**) and after (**2a**, **2b**, **3a**, **3b**) exposure to CA4+. Scale bars, 500 µm. **C** Color maps of representative contrast-enhanced μCT images of control and operated tibias at 1, 3, and 7 days post-operation, viewed from the transverse plane. The color scale corresponds to Hounsfield Units (HU). For reference, air attenuates at −1000 H and water at 0 HU. Scale bars, 500 µm. **D** Quantification of the injury gap volume using CECT imaging. Data are presented as mean ± SD, *n* = 5 mice for each time point (**d1**, **d3**, **d7**)). The asterisks indicate significant difference: Two-tailed Student’s unpaired samples *t* test; **p  =  0.0079 (**d1** vs **d7**). **E** SafraninO/Fast Green staining of control and operated tibias, 1 day (**a**, **b**), 3 days (**c**, **d**), and 7 days (**e**, **f**) after SH1-like surgery. Inset, central area of the defect (1–3). Scale bars, 100 µm. **F** ImageJ quantification of the injury. A is healthy cartilage area (SafraninO+), B is injured area (SafraninO−), and A+B is the total area. Percentage (%) cartilage regeneration is A/A+B. Data are presented as mean ± SD, *n* = 3 mice. The asterisks indicate significant difference: Two-tailed Student’s unpaired samples *t* test; ***p  =  0.000839 (**d1** vs **d7**), **p  =  0.007121 (**d3** vs **d7**), *p  =  0.037861 (**d1** vs **d3**). Complete statistical information is provided in Supplementary Table 1.

To investigate whether FoxA2+ cells expand and repair the GP cartilage in response to trauma, we developed a murine model of GP injury. Salter–Harris type1 fractures (SH1), which involve separation across the entire physis, through the HZ, have the best prognosis for repair and are the least likely to cause growth arrest^3,4^. We reproduced this clinical observation and developed a SH1-like surgical model, to test if FoxA2+ cells could repair the GP cartilage. A 30 g needle is passed in the transverse plane, through the HZ of the proximal tibial physis, creating a discrete defect, visualized by Contrast-Enhanced Computed Tomography (CECT)^17,18^ (Fig. 6A, B). Quantitative analysis of sequential sagittal plane images estimates that the defect tracks, on average, 2216 ± 195.6 μm medial to lateral, 280 ± 60 μm anterior to posterior, 165.6 ± 36.9 μm height, and 28.6° ± 5.5 angle relative to mid-coronal plane (Supplementary Fig. 8A, B). Color maps of representative CECT images of control and operated tibias reveal that injury site heals within 7 days, irrespective of mouse gender (Fig. 6C). The injury gap volume decreases significantly from 0.29 ± 0.07 mm^3^ at 1 day, to 0.04 ± 0.04 mm^3^, close to background level, at 7-day post-op (Fig.6D). Overall longitudinal growth was not affected at 7- and 21-day post-op (Supplementary Fig. 8C).

At 1-day post-op, the injured GP displays significant damage in the HZ (Fig. 6E(a, b)). In addition, given that the central region is more curvilinear, with a shorter GP in the middle, the injury causes more damage in this area, destroying the HZ, the PZ, and most of the RZ. At 1-day post-op, the healthy cartilage area is 42% of the total measured area (healthy plus injured) (Fig. 6F). Within days, the defect shows healing and the cartilage is regenerating. By the 3rd day, the central gap is closing in, and the healthy cartilage area is up to 63% ((Fig. 6E(c, d), F). By the 7th day, the GP is 96% regenerated, displaying all three layers (RZ, PZ, HZ) ((Fig. 6E(e, f), F). The injured GP healed with physeal cartilage, not fibrocartilage or bone.

### FoxA2+ cells expand and regenerate the GP after SH1-like injury

**Fig. 7:**
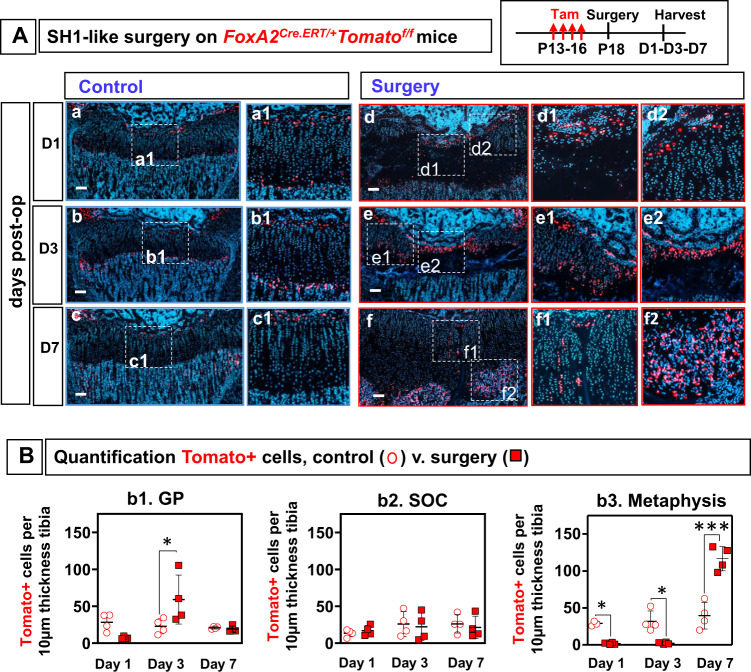
FoxA2+ cells expand in response to SH1 (Salter–Harris type 1)-like surgery. **A** Control (**a**–**c**) and operated (**d**–**f**) tibia from *FoxA2*^*CreERT2/+*^;*Tomato*^*fl/fl*^ mice, injected with tamoxifen P13-P16, operated via SH1-like surgery at P18, and harvested at 1-, 3-, 7-day post-op. Tomato fluorescence (red), Hoechst (blue). Insets, magnified view of injury (**d1**–**2**, **e1**–**2**, **f1**–**2**) or control (**a1**, **b1**, **c1**). Scale bars, 100 µm. **B** Quantification FoxA2+(Tomato+) cells at **d1**, **d3** and **d7** post-op in the GP (**b1**), SOC (**b2**), and metaphyseal bone (**b3**), in tibial sections from control (open circles) and operated (closed squares) limbs. The number of Tomato+ cells in the metaphyseal bone (MB) is counted in an area extending 100 µm away from the GP/MB interface, towards the MB. The number of Tomato+ cells in the SOC is counted in an area extending 100 µm away from the GP/SOC interface, towards the SOC. The number of Tomato+ cells in the GP is counted in an area extending 100 µm away from the GP/SOC interface, towards the GP. Each point represents an average of *n* = 3 sections collected from the lesion area, per hindlimb. Data are presented as mean ± SD of *n* = 4 mice. The asterisks indicate significant difference: One-way ANOVA, Tukey test; *p  =  0.032625 (Day 3: control vs surgery, GP), 0.03613 (Day 1: control vs surgery, Metaphysis), 0.021045 (Day 3: control vs surgery, Metaphysis), ***p  =  0.0000003 (Day 7: control vs surgery, Metaphysis). Complete statistical information is in Supplementary Table 1.

After SH1-like surgery on *FoxA2*^*CreERT/+*^*;Tomato*^*fl/+*^ mice, there was substantial expansion of Tomato+ cells in the injured GP (Fig. 7A, B, Supplementary Fig. 9). We quantified the number of FoxA2+ cells in GP cartilage as well as in neighboring metaphyseal bone (MB) and epiphyseal bone (SOC) (Fig. 7B). At 1-day post-op, there was no difference between GP FoxA2+ cells in control versus operated mice (Fig. 7A(a, d), Fig. 7B(b1)). However, there is a significant loss (94%) of FoxA2+ cells at the HZ/MB border, which continues to be sustained 3-day post-op (Fig. 7B(b3)). This indicates that there is no expansion of the MB Tomato+ cells in response to injury. Similarly, there is no expansion of the Tomato+ cells in the SOC at 1 day or 3 days after SH1-like surgery (Fig. 7B(b2)). However, there is a 2.7-fold expansion of Tomato+ cells in the GP 3 days after the SH1-like surgery (Fig. 7B(b1)).

These findings are supported by a proliferation assay, which demonstrates that Tomato+ cells are BrdU+ (Supplementary Fig. 10A). This expansion of FoxA2+ cells at 3 days post-op is the result of FoxA2+ cells labeled prior to surgery who are multiplying in response to injury, and not fresh labeling of FoxA2+ cells due to de novo expression of FoxA2 in the injured cartilage remnants. Immunohistochemistry for FoxA2 in the injured GP cartilage shows the expression of FoxA2 in its established RZ domain, but not in the cartilage remnants at d1, d2 or d3 post-op (a–c) (Supplementary Fig. 10B).

By 7-day post-op, FoxA2+(Tomato+) cells are significantly reduced in the GP tissue, and in parallel, we see a surge in Tomato+ cells in the MB (3-fold increase) (Fig. 7B(b3)). This suggests that the Tomato+ cells, that have expanded in the GP tissue at 3-day post-op, transited below in the bone spongiosa by 7-day post-op (Fig. 7A(f2)). In the center, where the wide gap was now reduced to a thin slit, the last Tomato+ cells are migrating down, aligned in short (4–8 cell) stacks of clones (Fig. 7A(f1)).

### FoxA2+ cells are needed for cartilage regeneration

**Fig. 8:**
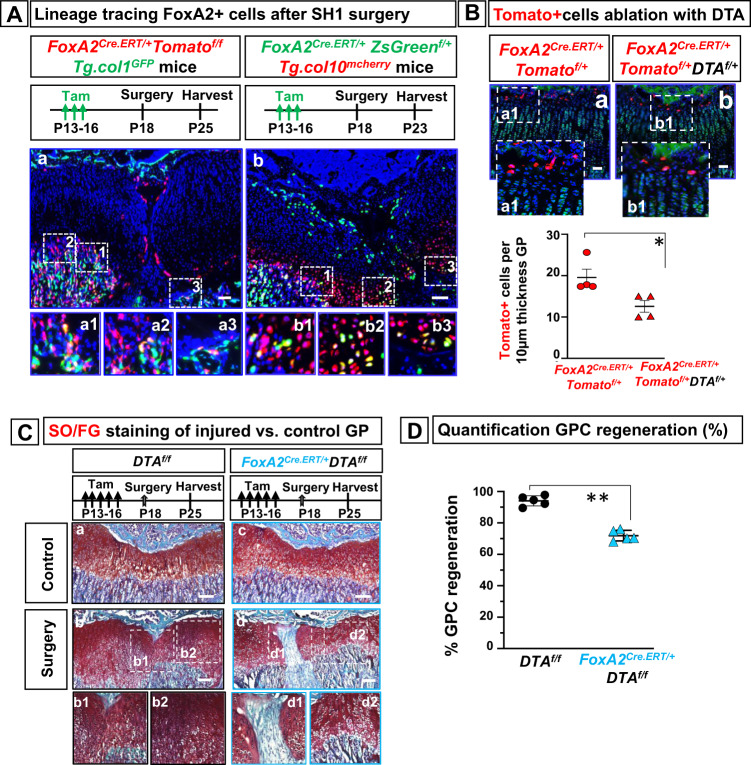
Ablation of FoxA2+ cells impairs cartilage regeneration. **A** Tibia sections from *FoxA2*^*CreERT/+*^*; Tomato*^*fl/+*^;*Tg.col1*^*GFP*^ mice treated with tamoxifen (Tam) P13-P16, operated via Salter–Harris type 1 (SH1)-like surgery at P18, harvested at 7 days post-op (**a**). Representative details from the metaphyseal bone in numbered insets (**a1**–**3**). Tibia sections from *FoxA2*^*Cre.ERT/+*^*; ZsGreen*^*f/+*^*;Tg.col10*^*mcherry*^ mice treated with tamoxifen P13-P16, operated via SH1-like surgery at P18, and harvested 5 days post-op (**b**). Representative details from the hypertrophic zone in numbered insets (**b1**-**3**). Scale bars are 100 µm. **B** Tibia sections harvested from *FoxA2*^*CreERT/+*^*;Tomato*^*fl/+*^ (**a**) and *FoxA2*^*CreERT/+*^*;DTA*^*f/+*^*;Tomato*^*fl/+*^ (**b**) mice treated with tamoxifen P13-P16, harvested at P18. Hoechst dye (blue), 5-DTAF (green). Insets, magnified view of the FoxA2+ cells visualized by Tomato fluorescence microscopy (red) (a1, b1). Scale bars 50 µm. Quantification of Tomato+ cells, per 10 µm tibial section of GP tissue, from *FoxA2*^*CreERT/+*^*; Tomato*^*fl/+*^ and *FoxA2*^*CreERT/+*^*; DTA*^*f/+*^*; Tomato*^*fl/+*^ mice. Data are presented as mean ± SD of *n* = 4 mice. The asterisks indicate significant difference: Two-tailed Student’s unpaired samples *t* test; *p  =  0.028571 (*FoxA2*^*CreERT/+*^*; Tomato*^*fl/+*^ vs *FoxA2*^*CreERT/+*^*; DTA*^*f/+*^*; Tomato*^*fl/+*^). **C** Safranin O/Fast Green staining of tibia sections from control *DTA*^*f/f*^ mice (**a**, **b**) and *FoxA2*^*CreERT/+*^*; DTA*^*f/f*^ mice (**c**, **d**), treated with tamoxifen P13-P16, operated at P18 and harvested 7 days after injury. Insets, magnified view of the injury callus in *FoxA2*^*CreERT/+*^*; DTA*^*f/f*^ mice (**d1**, **d2**), or *DTA*^*f/f*^ mice (**b1**, **b2**). Scale bars are 100 µm. **D** Image J quantification of GP cartilage regeneration after surgery in *FoxA2*^*CreERT/+*^*; DTA*^*f/f*^ mice compared with *DTA*^*f/f*^ mice. Percentage (%) cartilage regeneration is calculated by dividing the area of SafraninO+ GP cartilage to the total GP cartilage area (injured plus healthy). Data are presented as mean ± SD, *n* = 5 mice. The asterisks indicate significant difference: Two-tailed Student’s unpaired-samples *t* test; **p  =  0.007937 (*FoxA2*^*CreERT/+*^*; DTA*^*f/f*^ vs *DTA*^*f/f*^). Complete statistical information is provided in Supplementary Table 1.

To determine whether FoxA2+ cells participate in regenerating the injured GP tissue via multi-lineage differentiation, we asked if FoxA2+ cells differentiate into col.10+ cells (chondrogenic lineage) or col.1+ cells (osteogenic lineage). SH1-like surgery on *FoxA2*^*Cre.ERT/+*^*;ZsGreen*^*f/+*^*;Tg.col10*^*mcherry*^ mice demonstrates that FoxA2+(ZsGreen+) cells generate yellow hypertrophic cells (col.10^mcherry^+) (Fig. 8A(b)), while SH1-like surgery on *FoxA2*^*Cre.ERT/+*^*;Tomato*^*f/f*^*;Tg.col1*^*GFP*^ mice shows that FoxA2+(Tomato+) cells transit in the metaphyseal bone to become yellow bone cells (col.1^GFP^+) (Fig. 8A(a)).

To inquire whether FoxA2+ cells are needed for cartilage regeneration, we generated *FoxA2*^*CreERT/+*^*;DTA*^*f/f*^ mice, in which FoxA2+ cells were ablated by production of diphtheria toxin (DTA). In *FoxA2*^*CreERT2/+*^*;DTA*^*f/+*^*;Tomato*^*fl/+*^ mice there was a 36% decrease in FoxA2+(Tomato+) cells compared with *FoxA2*^*CreERT2/+*^*;Tomato*^*fl/+*^ littermates (Fig. 8B). Loss of FoxA2+ cells lead to a delay in cartilage closure in *FoxA2*^*CreERT2/+*^*;DTA*^*f/f*^ mice 7-day post-op (Fig. 8C(b1, d1)). Cartilage regeneration in *FoxA2*^*CreERT2/+*^*;DTA*^*f/f*^ mice was significantly reduced (down to 72%) compared with control mice (Fig. 8C, D), suggesting that FoxA2+ cells are necessary for GP repair.

## Summary

**Fig. 9:**
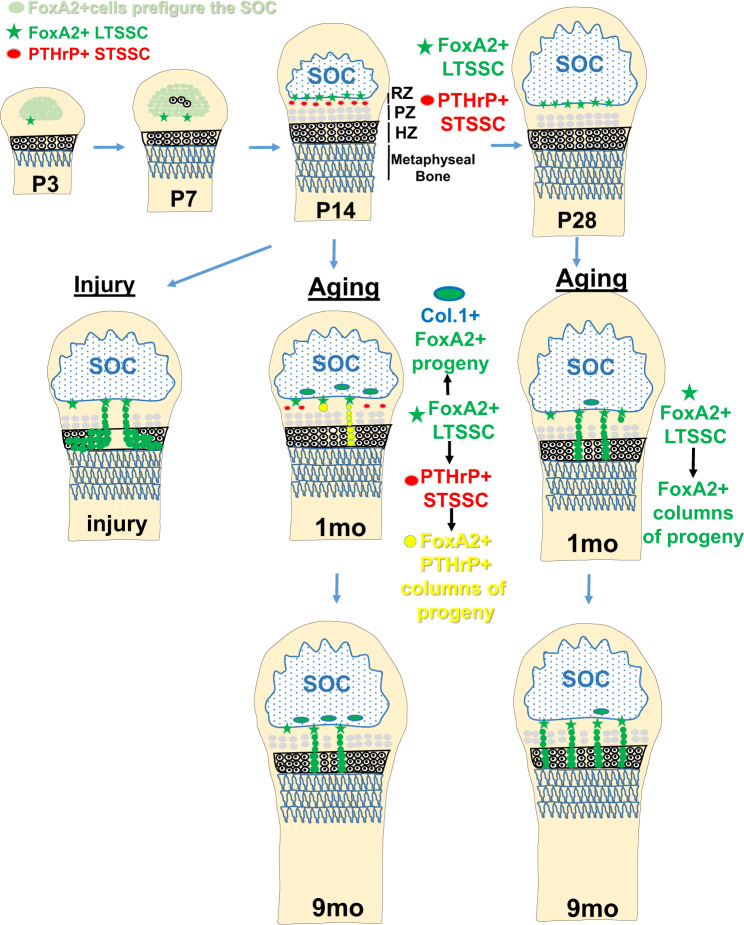
FoxA2+ LTSSC maintain cartilage homeostasis by contributing to long-term self-renewal, chondrogenic differentiation and cartilage repair after injury. FoxA2+ LTSSC, residual from epiphyseal cartilage postnatal development, located on the outskirts of the SOC, contribute to the top compartment of the RZ. FoxA2+ cells are distinct from the short-term PTHrP+ stem cells located at the bottom of the RZ. Prior to P28, FoxA2+ cells have a dual osteo-chondro-progenitor fate, contributing to the chondrogenic lineage (by forming columnar stacks of chondrocytes in the GP), and the osteogenic lineage (by forming col.1+ bone cells in the SOC). After P28, FoxA2+ LTSSC remain mostly in GP, and their contribution to the SOC is reduced. GP injury activates FoxA2+ cells to undergo proliferative expansion and to provide a framework for the regenerating tissue. In conclusion, FoxA2+ cells, located in the top compartment of the RZ, are a PTHrP-(negative) long-term skeletal stem cell (LT-SSC) population necessary for both GP turnover and cartilage regeneration following injury. SOC = secondary ossification center, RZ = resting zone, PZ = proliferating zone, HZ = hypertrophic zone.

In summary, we demonstrate that a population of FoxA2+col10-cells, residual from epiphyseal cartilage postnatal development, contributes to the top sub-compartment of the RZ (Fig. 9). Based on their location, FoxA2+ cells are physically separated from the PTHrP+ cells, which are located at the bottom of the RZ, and exhibit higher clonogenicity and longevity than PTHrP+ cells. Short-term pulse-chase of the FoxA2+ cells generates dyads/clusters of cells, whereas long-term pulse-chase generates increasing columns of proliferating progeny, suggesting that FoxA2+ cells may first give rise to progeny that subsequently differentiate into columns of chondrocytes. Prior to P28, FoxA2+ cells have a dual osteo-chondro-progenitor fate, and after P28, FoxA2+ LTSSC remain mostly in GP, while their contribution to the SOC winds down significantly. In a murine model of GP injury, FoxA2+ cells expand in response to trauma and participate in production of physeal cartilage allowing for successful regeneration. In conclusion, FoxA2+ cells are a LTSSC population necessary for both GP turnover and cartilage regeneration following injury.

## Discussion

While POC (primary ossification centers) of the long bones form at E15.5, during mouse embryonic development, the SOCs appear sometime later, primarily after birth, around postnatal day P7^19,20^. Chondrocyte hypertrophy (P7-P8) is followed by osteogenesis (P9-P10) emerging in the center of the epiphysis and by SOC enlargement and maturation thereafter^19^. A rise in serum thyroid hormone (TH) level during the murine prepubertal growth period initiates SOC activity, by promoting conversion of Osterix-expressing chondrocytes into bone matrix producing osteoblasts via a hedgehog signaling pathway^19^.

Our findings provide experimental evidence that SOC formation starts earlier than previously thought. Lineage tracing shows that the cells fated to become SOC are marked by FoxA2 expression as early as P0, and are morphologically indistinguishable from the columnar GP cells before P5. Since the first col10+ hypertrophic chondrocytes appear by P7, FoxA2 expression prefigures SOC formation, before overt chondrocyte differentiation. These findings are consistent with the role of FoxA factors as pioneer transcription factors, able to bind nucleosomal covered DNA and to disrupt local chromatin structure prior to gene transcription^21–23^. This FoxA factors-chromatin interaction facilitates binding of other transcription factors including C/EBP, GATA4, Nf1, androgen and glucocorticoid receptors to adjacent binding sites^22,24,25^. Thus, it is possible that FoxA factors provide competence for chondrocyte hypertrophy by promoting interaction with other transcription factors.

At the interface between SOC and GP, a population of FoxA2+ cells builds up in the GP cartilage during early postnatal skeletal development. These FoxA2+ cells are left behind from epiphyseal cartilage postnatal development (during SOC prefiguration, hypertrophy, and enlargement), and give rise to the GP RZ top layer. At 3 months after the initial labeling, FoxA2+ cells are still present in the GP and give rise to columns of progeny. By 9 months, the number of columns significantly increases, indicating that the FoxA2+ cells increasingly contribute to GP cartilage over time. In addition, FoxA2+ cells can also give rise to col.1+bone cells in the SOC during early postnatal development. However, it is unclear whether the same FoxA2+ cell has a dual fate, or the FoxA2+population is heterogeneous, with some cells destined for osteogenic lineage, and others for the chondrogenic lineage.

After P28, FoxA2+ cells remain mostly in GP, and their contribution to the SOC slows down significantly. This shift in clonality coincides with the maturation of the SOC into epiphyseal bone, as previously reported by Newton et al. who reveal a significant role for the SOC in the renewal of GP chondroprogenitors by providing a stem cell niche^10^. Newton et al. describe at least two sub-populations of stem cells, with different proliferation rates, similar to stem cell niches in hair follicles^26,27^, bone marrow^28^ and intestine^29^. In addition to the sub-population dedicated to making columnar chondrocytes, there seems to be another, more dormant, sub-population of LRC (label-retaining cells)^10^. Even after 1–2 months of tracing, some LRCs remained at the top of the RZ, either as single cells, or as small clusters, but not columns of progeny. This more quiescent group is primarily located next to the epiphyseal bone and the SOC, reinforcing the idea of the SOC as a center of signaling that guides, in some –not yet fully understood- way, the stemness of the RZ cells^10^. Our experiments support these previous findings and show that FoxA2+ cells constitute this more quiescent, LTSSC population, located at the border between GP and the SOC.

FoxA2+ cells are mutually exclusive with the previously characterized PTHrP+ stem cells^11^, and exhibit higher clonogenicity and longevity. Mizuhashi et al. noted higher longevity for *PTHrP*^*CreERT2/+*^*;Tomato*^*fl/+*^ clones, but the labeling involves an early-pulse (postnatal day6) and a long-chase, which caused extensive marking of PTHrP+ descendants in the RZ^11^. Some labeled cells reach as high as the FoxA2+ expression domain, next to the newly formed SOC. Thus, this broad labeling may include some of the FoxA2+ cells, hence the LTSSC characteristics of some established clones. In contrast, we demonstrate that *PTHrP*^*mcherry*^ cells exhaust within five passages in a cfu assay, unlike FoxA2+ cells, which maintain the colony forming ability beyond nine passages, demonstrating long term self-renewability.

In addition to demonstrating that FoxA2+ stem cells retain long-term self-renewability in vitro, we also found that FoxA2+ cells preserve self-renewal ability and multipotency in a serial transplantation assay. Published reports indicate that FoxA2 serves as a stemness regulator, controlling the self-renewal of ovarian cancer stem cells^30^, and it is required for proper chromatin remodeling during human pluripotent stem cells differentiation to pancreatic progenitors^31^. It will be interesting to establish whether FoxA2-dependent transcription is required for FoxA2+ LTSSC self-renewability.

Salter–Harris classification system divides GP fractures into five groups predicated on fracture morphology involving the physis ± epiphysis and/or metaphysis and the deleterious effect on appendicular growth^3^. Salter–Harris fractures through the physis often result in the formation of a “bony bar” bridging the epiphysis to the metaphysis^32–34^. In contrast, Salter–Harris type1 fractures where the cleavage plane is along the GP, preserving the RZ, heal without significant growth disturbance^3,4^. Thus, we developed a surgical model that emulates SH1 fracture morphology by creating a discrete defect through the HZ of the physis. Within days, FoxA2+ cells expand in response to injury allowing for regeneration of physeal cartilage and maintenance of longitudinal bone growth. This is consistent with previous published reports where removal of PZ and HZ from the GP allowed for GP regeneration, as long as the RZ is preserved^5,6^. However, when the GP was progressively reduced to the RZ top compartment, regeneration of the GP took longer^5^. This supports our findings that FoxA2+ cells, located in the RZ top compartment, behave as LTSSC, first giving rise to progeny, which then produce columns of chondrocytes. These results are relevant for understanding the pathophysiology of GP injuries in children and deriving treatment strategies to prevent subsequent disturbances to symmetric, longitudinal bone growth that can cause disability in adulthood^2^.

## Methods

Mouse strains^12,35–39^: *FoxA2*^*Cre.ERT/+*^ mice^12^ (JAX stock#008464)*, Agg*^*Cre.ERT/+*^ mice (JAX stock#019148)*, Tomato*^*fl/fl*^ mice^36^ (JAX stock#007914), *ZsGreen*^*fl/fl*^ mice^36^ (JAX stock #007906), *DTA*^*f/f*^ mice^39^ (JAX stock#009669), *Tg.col1*^*gfp*^ mice^35^ (JAX stock #013134), *PTHrP*^*mcherry*^ mice^38^ (JAX stock#032872) and *Tg.col10a1*^*mCherry*^ mice^37^ (JAX stock#017465). All genetically modified mouse lines used in this study were backcrossed to a C57Bl/6J background. Mice with both sexes (males and females, randomized in groups) were used up to 1 year of age. The animals were housed in Northeastern University accredited facilities with free access to standard rodent chow and water, with a controlled 12 h light-dark cycle. Intraperitoneal tamoxifen (100 mg/kg/day) injection was given daily to activate CRE expression as indicated to generate time-dependent and tissue-specific mutant mice. All studies were carried out using littermate cohorts. All procedures involving mice were performed with approved protocols from Northeastern Institutional Animal Care and Use Committee (IACUC). For BrdU labeling, mice were administered 3 injections (1 ml/100 g body weight, Cat#000103, Invitrogen, USA) at 12 h intervals before harvest and sacrificed 2 h after the last injection.

Isolation of GP cells. GP cartilage was dissected out of proximal tibia, distal radius and distal ulna of P18 *FoxA2*^*CreERT/+*^*;ZsGreen*^*fl/+*^*;Tg.col10*^*mCherry*^ mice or *PTHrP*^*mcherry*^ mice that received intraperitoneal tamoxifen, 100 mg/kg/day (Cat# T5648, Sigma, USA) at postnatal days P13-17. The surrounding tissues adhering to the growth plate were removed and the growth plate cartilage was subjected to predigestion with 0.2% Collagenase P (Cat# 11213857001, Sigma-Aldrich, USA) in DMEM high glucose (Cat# 11885084, Life Technologies, ThermoFisher, USA) in a shaking waterbath at 37 °C, 150 rpm for 40 min. The predigested growth plates were cut into 1–2 mm pieces with a sterile scalpel and subjected to final digestion with enzymes, 0.1 % collagenase II (Cat# 17101-015, Life Technologies, ThermoFisher, USA) and 0.05 % trypsin (Cat# 25200-072, ThermoFisher, USA) at 37 °C in a CO_2_ incubator for 4 h. After complete digestion, the cell suspension was passed through a 70 μm cell strainer, centrifuged at 1000 × *g* for 20 min and the pellet was resuspended in complete growth medium (DMEM containing high glucose (4.5 g/liter), 1% l-glutamine, 10% fetal bovine serum, 100 U/ml of penicillin, and 100 mg/ml of streptomycin).

Flow cytometry and cell sorting. For Fluorescence-activated cell sorting (FACS), on average 500,000 cells (from 5 to 6 mice) were washed 2x with 2% heat-inactivated fetal calf serum in PBS, and then resuspended in PBS. The analysis and sorting for ZsGreen or mcherry were performed with BD FACS Aria II equipped with six laser system 355, 405, 445, 488, 561, and 641 nm. FACS images show the representative contour plots derived from the analysis of three independent litters.

Real-time PCR analysis. Real-time PCR was performed using TaqMan^TM^ Fast Advanced Master Mix (Cat#4444553, Applied Biosystems, USA). All quantitative gene expression data were normalized to the expression levels of PPIB. The TaqMan probe ID were Mm00545794 (Aggrecan), Mm01309565 (COL2A1), Mm00801666 (COL1A1), Mm00475834 (ALP) and Mm00478295 (PPIB) purchased from ThermoFisher.

Colony Forming Unit Assays. FoxA2+col.10- (green) cells, FoxA2+col.10+(yellow) cells, or PTHrP+ cells were seeded at a density of 10^3^cells/10 cm culture dishes or 5–10 cells/well into 96-well plates. They were incubated for 21 days in a complete growth medium, with media change every 4–5 days. Cloning cylinders (Cat# 3166-6, Corning Incorporated, USA) were positioned around each single colony for trypsinization of cells. Progeny cells from each colony underwent serial passages after seeding at the same density for colony forming unit assay as mentioned above for the parent cells.

Trilineage differentiation. Osteogenesis was induced as described previously^40,41^. Cells were seeded in triplicates in 12-well plates (5 × 10^4^ cells/well) and grown for 24 h in complete growth medium. To induce osteoblast differentiation, cells were cultured in osteogenic medium (Minimum Essential Medium alpha (MEM α, Cat# 12571-048, ThermoFisher, USA) supplemented with 1% L-glutamine, 10% fetal bovine serum, 10 mM β-glycerophosphate (Cat# G9422, Sigma-Aldrich, USA) and 50 μg/ml ascorbic acid) for 4 weeks. Osteogenic nodules formed after differentiation were stained by Alizarin Red-S (Cat# A5533, Sigma-Aldrich, USA) staining as described previously^40,42^ and visualized using an inverted light microscope. For adipogenesis, cells were treated with MEM α supplemented with 1% L-glutamine, 10% fetal bovine serum, 60 μM Indomethacin (Cat# 73942, Stemcell Technologies, Canada), 0.1 μM Dexamethasone (Cat# D4902, Sigma-Aldrich, USA) and 50 μg/ml ascorbic acid(Cat# A5960, Sigma-Aldrich, USA) for 4 weeks as described before^40,41^. Neutral lipid deposits in differentiated adipocytes were stained by LipidTOX red neutral lipid fluorescent stain (Cat# H34476, Invitrogen, USA). Chondrogenesis was induced by culturing cell pellets in StemXVivo chondrogenic base media (CCM005, R&D Systems, USA) supplemented with StemXVivo chondrogenic supplement (CCM006, R&D Systems, USA) according to procedures described by manufacturer’s instructions. After 28 days, the proteoglycan deposits in the cartilage matrix were stained by Alcian-Blue 8GX (Cat# A5268, Sigma-Aldrich, USA) staining as described previously^43^ and visualized using an inverted light microscope.

In vivo serial transplantation of cells. FoxA2+(ZsGreen+) cells isolated from *FoxA2*^*Cre.ERT/+*^ *ZsGreen*^*f/+*^ *Tg.col10*^*mcherry*^ mice were expanded in culture for 30 days. The cells were then collected, labeled with DiD lipophilic dye (Invitrogen, Cat# V22887) and resuspended in Matrigel (Corning Life Sci., Cat# 356255) at 4 °C resulting in a final cell concentration of 10^5^ cells/200 µL that was injected subcutaneously into Zsgreen- littermates. Injected zsGreen+ cells developed into a graft after 30 days. The grafts were surgically removed for cell isolation and DiD analysis (by flow cytometry) to monitor cell proliferation by dye dilution. To determine if cells retained self-renewal ability after expansion in the recipient mice, the FoxA2+ cells isolated from each graft were examined for colony forming abilities under culture conditions. For serial transplantation, FoxA2+ stem cells isolated from the grafts of secondary recipients were re-stained with DiD, re-transplanted into a tertiary recipient, allowed to develop in vivo for 30 days, after which they were tested for cell proliferation (by DiD fluorescence analysis) and for colony forming abilities in culture. To determine, if cells retained multi-lineage differentiation capabilities at the end of serial transplantation, the ZsGreen+ cells isolated at the end of serial transplantation assays were lineage-primed under chondrogenic, adipogenic or osteogenic conditions, for 3 days, prior to subcutaneous transplantation in ZsGreen^−^ littermates. After 30 days, the grafts were surgically removed, fixed in 10% neutral buffered formalin, and cryoprotected in 30% sucrose solution before freezing in Optimal cutting temperature (OCT) compound blocks. Cryostat sections from the blocks were stained using Alcian blue, LipidTox or Alizarin Red-S staining procedures for examining differentiated cell types.

SH1-like surgery. A SH1-like injury model was established by inducing a transverse excisional biopsy in C57B6 mice, across the lower region (HZ) of proximal tibial GP, using a 30 g needle. Surgical manipulations in mice were performed under general anesthesia by isoflurane (Cat#NDC14043-704-05, Patterson Veterinary, Greeley, USA) inhalation at ~4% (v/v) for induction and at ~2.5% (v/v) for maintenance, which was delivered with oxygen through nose cone for 15–20 min. Prior to surgery, the animals’ knees were shaved, and treated with betadine and alcohol (alternating 3 times). The joint capsule immediately medial to the patellar tendon was incised and opened with micro Iris scissors. A 30 g needle (0.312 mm outer diameter) was passed, in the transverse plane, through the lowest part of murine proximal tibial physis. The needle advances anteromedial to posterolateral, creating a discrete cylinder-shaped hole through the HZ of the physis. Control mice are sham-operated mice. The joint capsule was closed with a continuous 5–0 Vicryl suture (Cat#J495, Ethicon Inc., NJ).

Histology. For histological staining, hindlimbs were fixed in 10% neutral buffered formalin solution, decalcified in 10% EDTA, frozen-embedded and sectioned at 10 µm, then stained with Hematoxylin /Eosin (Sigma, cat# GHS216), SafraninO/Fast Green^44^ (SafraninO, Cat#S2255; Fast Green, Cat# F7252, Sigma-Aldrich, USA), 5-(4,6-Dichlorotriazinyl) Aminofluorescein (5-DTAF)(Cat# D16, ThermoFisher, 0.001% in PBS), Hoechst dye (Cat# H3570, Life Technologies Corporation, 1:2000 in PBS).

Histomorphometry. Histomorphometry was performed using ImageJ image processing and analysis software (Version 1.6.0_24; Java, National Institute of Health, USA). For SH1 surgery, we calibrated the images by using a global scale bar of 100 μ*m*. ImageJ quantification of the SH1-like injury involves measurement of Area A as SafraninO+ area of healthy cartilage, Area B as the injured area, characterized by Safranin O loss, and Area A + B as the total area. Percentage (%) regeneration is calculated by dividing the area of SO+healthy GP cartilage (A) to the total GP area (A + B) from *n* = 3 sections collected from the lesion area, per hindlimb. Quantification FoxA2+(Tomato+) cells at d1, d3 & d7 post-op in the metaphysis, SOC and GP was performed in tibial sections from control and operated limbs. The number of Tomato+ cells in the metaphyseal bone (MB) is counted in an area extending 100 µm away from the GP/MB interface, towards the MB. The number of Tomato+ cells in the SOC is counted in an area extending 100 µm away from the GP/SOC interface, towards the SOC. The number of Tomato+ cells in the GP is counted in an area extending 100 µm away from the GP/SOC interface, towards the GP. Each point represents an average of *n* = 3 sections collected from the lesion area, per hindlimb.

Clone number quantification. To quantify the number of FoxA2+(ZsGreen+) cells present in the GP, during early postnatal development, we counted the number of FoxA2+ cells located in GP (extending 100 µm away from the GP/SOC interface, towards the GP). To calculate the percentage (%) cells in the GP v. SOC, we divided the number of Tomato+ cells in the GP (outside the green 5-DTAF domain) by the total number of Tomato+ cells (located in both GP and SOC). The number of Tomato+ cells represents a sum of *n* = 8 sections per mouse hindlimb. Percentage (%) cells in the SOC was calculated by dividing the number of Tomato+ cells in the SOC (inside the green 5-DTAF domain) by the total number of Tomato+ cells (located in both GP and SOC). To assess the ability of FoxA2+ cells to give rise to descendants over time, we quantified the number of FoxA2+ single cells, clusters and columns at 1 day, and 1, 3, 9 months after labeling. Each point represents the sum of *n* = 8 sections per mouse hindlimb. Percentage (%) single cells was calculated from the ratio of number of single Tomato+ cells in the GP (outside the green 5-DTAF domain) to the total number of Tomato+ units (single cells, clusters and columns). One Tomato+ unit, is defined by 1 single cell, or 1 cluster comprised of two or more cells grouped together, or by a column of cells. Same calculations were performed for clusters and columns of progeny. Quantification of tamoxifen labeling efficiency was performed by calculating the number of FoxA2+ cells detected via IHC or by Tomato+ fluorescence per 10 µm thickness section, as an average of *n* = 4 sections. The number of FoxA2+ cells in the GP is counted in an area extending 100 µm away from the GP/SOC interface, towards the GP.

Immunohistochemistry. For immunohistochemistry, we used frozen-embedded sections. The following antibodies were used: FoxA1 (Abcam, Cat#ab23738, 1:500), FoxA2 (Millipore, Cat#07-633, 1:2000), FoxA3 (Santa-Cruz, Cat#sc-25357, 1:50), Ki67 (Cell Signaling, Cat#9129S, 1:800), BrdU (ThermoFisher, Cat# B35138, 1:100). The sections were incubated with Dual Endogenous Enzyme Block (Dako, cat#S200380) for 30 min at room temperature in order to suppress endogenous alkaline phosphatase and peroxidase enzymes. Blocking was performed in 0.5% TNB buffer (PerkinElmer, cat#FP1020) for 1 h. The samples were washed with TBST (TBS with 0.05% TWEEN® 20 (Sigma, cat#P1379)) and incubated with anti-rabbit IgG horseradish peroxidase (PerkinElmer, cat#NEF812001EA), for 45 min at room temperature. After washing the samples in TBST, the slides were incubated for 5 min in a tyramide-biotin working solution, which was prepared in a 1:50 dilution using the TSA™ Biotin System (PerkinElmer cat#NEL700A001KT). For detection, the samples were incubated in strepdavidin conjugated with Alexa Fluor 568 dye, diluted 1:500 in 0.5% TNB buffer, for 1 h in the dark at room temperature. Lastly, the samples were mounted using ProLong™ Diamond Antifade Mountant with DAPI (Invitrogen, cat# P36966).

CECT and X-ray Imaging and Analysis. Non-contrast-enhanced μCT cannot directly visualize unmineralized tissues such as cartilage, owing to the low x-ray attenuation coefficient of these tissues. To counteract this, we immersed the samples in CA4+, an iodinated, cationic contrast agent, that is electrostatically attracted to the anionic glycosaminoglycans (GAG) of the cartilage ECM. The X-ray attenuation induced by the CA4+ is highly correlated with the fixed negative charge density of the tissue and is thereby a reliable tool for amplifying attenuation of GAG-rich tissues such as cartilage. Contralateral and surgical mouse tibial explants from 1, 3, 7, and 21 days post-surgery were positioned in airtight CT vials. Baseline CT slices were acquired transaxially at 18 μm resolution, 70 kVp tube voltage, 114 mA, 300 ms integration time (μCT40, SCANCO Medical AG, Brüttisellen, Switzerland). Samples were then immersed in CA4 + solution (12 mgI/mL, pH 7.4, 350–400 mOsm, 24 h) prior to CECT imaging using the same settings and μCT scanner. The μCT data was then converted to DICOM format. The GP cartilage was segmented from bone and surrounding soft tissues in a semi-automated process in order to obtain cartilage thickness, lesion dimensions, and lesion volume (Analyze™, AnalyzeDirect, Overland Park, KS). Select images were converted to color maps to enhance visualization of the GP cartilage. For X-ray Imaging and Analysis, tibial explants from 1, 3, 7, and 21 days post-operation were X-rayed at 75kVp, 6 min exposure time (Faxitron X-ray Cabinet Series, Model No. 43855 A, Faxitron X-ray Corporation, Wheeling, IL). The film was developed, and calipers were used to measure tibial length for each specimen.

Statistics and Reproducibility. Data were expressed as mean ± standard deviation (SD). Statistical analyses were performed using GraphPad Prism. Experiments are performed in triplicate, unless otherwise noted in figure legends. Comparisons between two independent groups were performed using an unpaired Student’s *t* test. Comparisons between multiple groups were performed using a one‐way analysis of variance (ANOVA) with Tukey’s post hoc analysis. A *p*-value below less than 0.05 was considered statistically significant. Single asterisks denote *p* < 0.05 (*p  <  0.05); Double asterisks denote *p* < 0.01 (**p  < 0.01); Triple asterisks denote *p* < 0.001 (***P < 0.001).

### Reporting summary

Further information on research design is available in the Nature Research Reporting Summary linked to this article.

## Supplementary information

Supplementary Information

Reporting Summary

## Data Availability

The data generated in this study (all Figures and Supplementary Figures) are provided as a Source Data file. Source data are provided with this paper.

## Source data

Source Data
