# Supplementary material for: A FoxA2+ long-term stem cell population is necessary for growth plate cartilage regeneration after injury
Source: Nat Commun. 2022 May 6;13:2515. doi: 10.1038/s41467-022-30247-1 (PMC9076650; doi:10.1038/s41467-022-30247-1)
Supplement: Supplementary file 1 — Supplementary Information [file 41467_2022_30247_MOESM1_ESM.pdf]

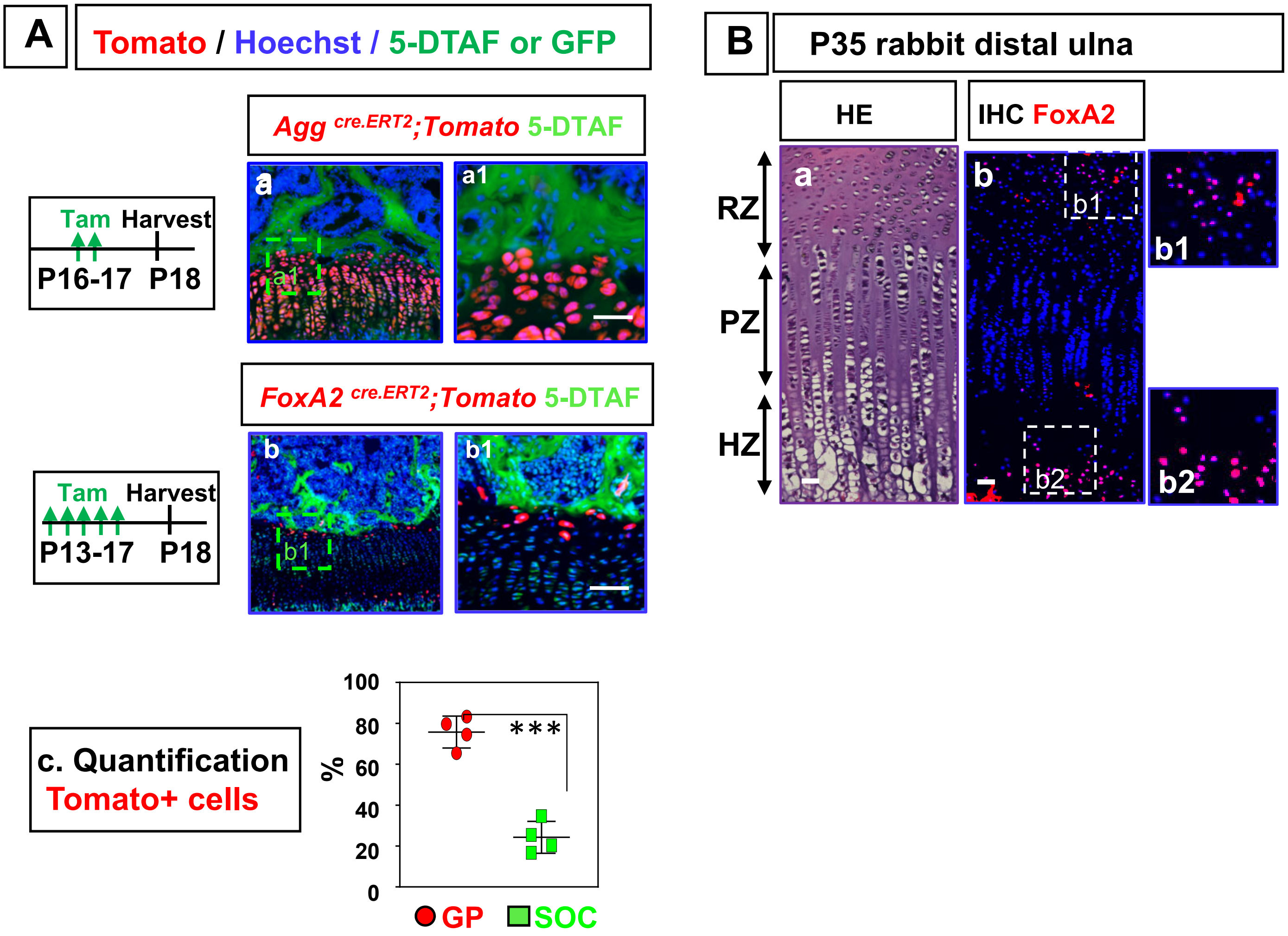

<u>Supplementary Fig. 1</u> (A) FoxA2+cells are located at the top of the RZ in both mice and rabbits.
(A) Tibia sections from $Aggrecan^{CreERT2/+}$;$Tomato^{fl/+}$mice, treated with tamoxifen (Tam) P16-P17, harvested at P18. Fluorescence microscopy for Tomato (red), Hoechst (blue) and 5-DTAF (green) (a). Tibia sections from $FoxA2^{CreERT2/+}$;$Tomato^{fl/+}$ mice treated with tamoxifen P13-P17, harvested at P18. Fluorescence microscopy for Tomato (red), Hoechst (blue) and 5-DTAF (green). Scale bars, 50μm (b). Percentage (%) cells in the GP vs. SOC. Percentage (%) cells in the GP is calculated by dividing the number of Tomato+ cells in the GP (outside the green 5-DTAF domain) to the total number of Tomato+ cells (located in both GP and SOC). Percentage (%) cells in the SOC is calculated by dividing the number of Tomato+ cells in the SOC (in the green 5-DTAF domain) to the total number of Tomato+ cells (located in both GP and SOC). Data are presented as mean±SD of n=4 mice. The asterisks indicate significant difference: Two-tailed Student's unpaired-samples t test; ***p = 0.000082 (GP vs SOC). Complete statistical information is provided in Supplementary Table 1 (c). **(B)** Hematoxylin and eosin (HE) staining (a), and immunohistochemistry (IHC) with anti-FoxA2 antibody (b) on P35 rabbit ulna. FoxA2 (red), and Hoechst dye (blue). RZ=Resting zone, PZ=Proliferating zone, HZ=hypertrophic zone. Scale bars, 50μm.

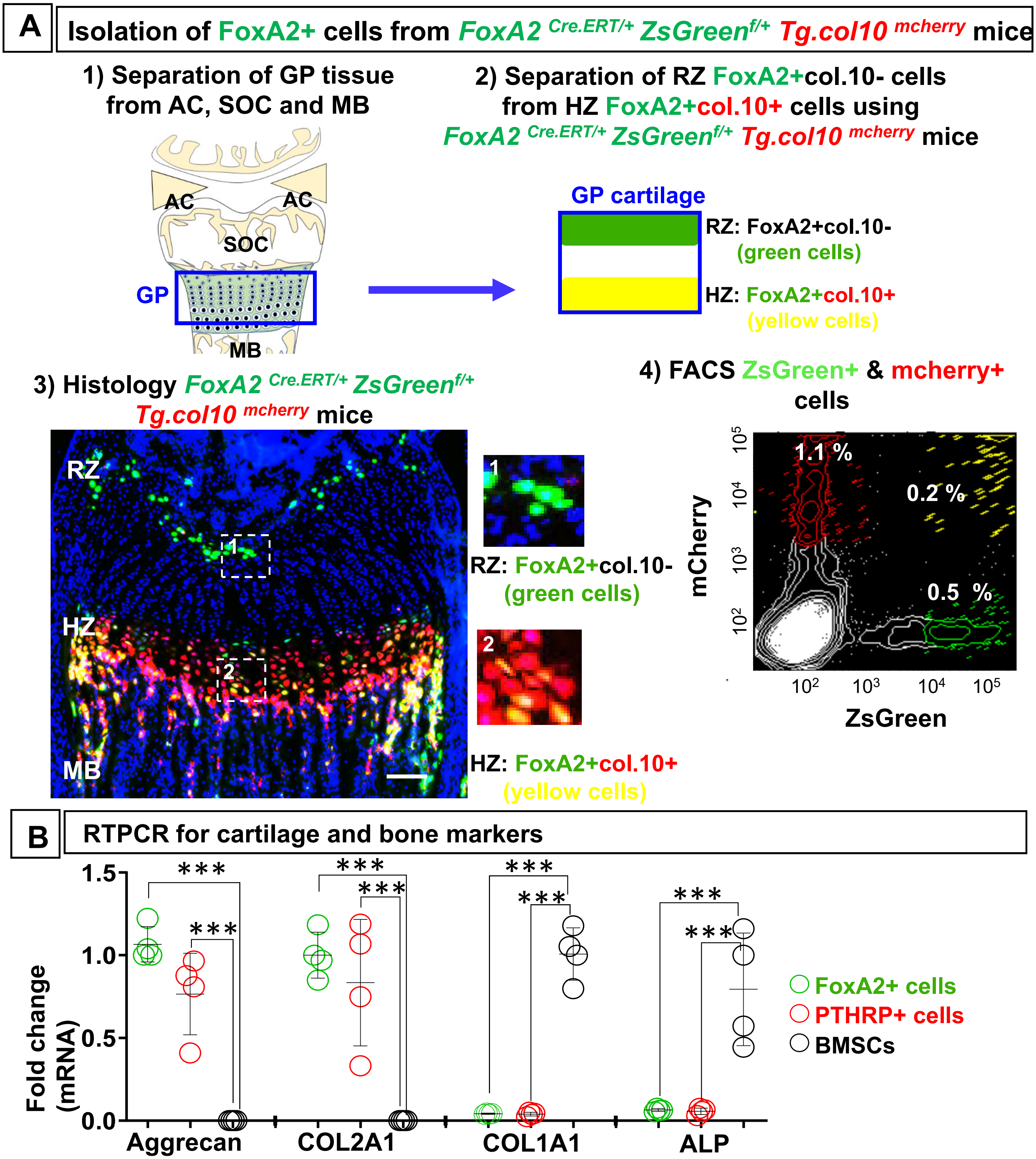

<u>Supplementary Fig.2</u> **Separation of RZ FoxA2+ (green) cells from HZ FoxA2+col.10+ (yellow) cells, isolated from** ***$FoxA2^{CreERT2/+}$;$ZsGreen^{fl/+}$;$col.10^{mcherry}$*** **mice. (A)** Schematics of GP tissue isolation. AC=articular cartilage, SOC=secondary ossification center, MB=metaphyseal bone, GP=growth plate (1&2). Tibia sections harvested from *$FoxA2^{CreERT2/+}$;$ZsGreen^{fl/+}$;$col.10^{mcherry}$* mice treated with tamoxifen P13-P17, harvested at P18, and counterstained with Hoechst (blue). RZ specific FoxA2+col.10-cells (green), HZ specific FoxA2+col.10+ cells (yellow), HZ specific col.10+ cells (red). RZ=resting zone, HZ=hypertrophic zone. Scale bars, 100µm. (3). Fluorescence-activated cell sorting (FACS) of GP cells isolated from forelimbs and hindlimbs of *$FoxA2^{CreERT2/+}$;$ZsGreen^{fl/+}$;$col.10^{mcherry}$* mice, injected with tamoxifen from P13 to P17, and harvested at P18. Separation of FoxA2+ (ZsGreen+) cells from col.10+ (mcherry+) cells (4). cont. next page.

Supplementary Fig.2 Cont.
(B) Cartilage and bone markers expression in RZ FoxA2+cells, PTHrP+cells and BMSCs (bone marrow mesenchymal cells). RTPCR for *Aggrecan, collagen 2, collagen 1, and alkaline phosphatase (ALP).* The mRNA fold-change is presented as mean±SD of n=4 samples. The asterisks indicate significant difference: One-way ANOVA, Tukey test; ***p = 2.61879E-08 (Aggrecan: FoxA2+ cells vs BMSCs), 3.09872E-05 (Aggrecan: PTHRP+ cells vs BMSCs), 1.1691E-07 (COL2A1: FoxA2+ cells vs BMSCs), 5.89962E-06 (COL2A1: PTHRP+ cells vs BMSCs), 2.65003E-07 (COL1A1: FoxA2+ cells vs BMSCs), 2.51267E-07 (COL1A1: PTHRP+ cells vs BMSCs), 7.20261E-05 (ALP: FoxA2+ cells vs BMSCs), 6.00535E-05 (ALP: PTHRP+ cells vs BMSCs). Complete statistical information is provided in Supplementary Table 1.

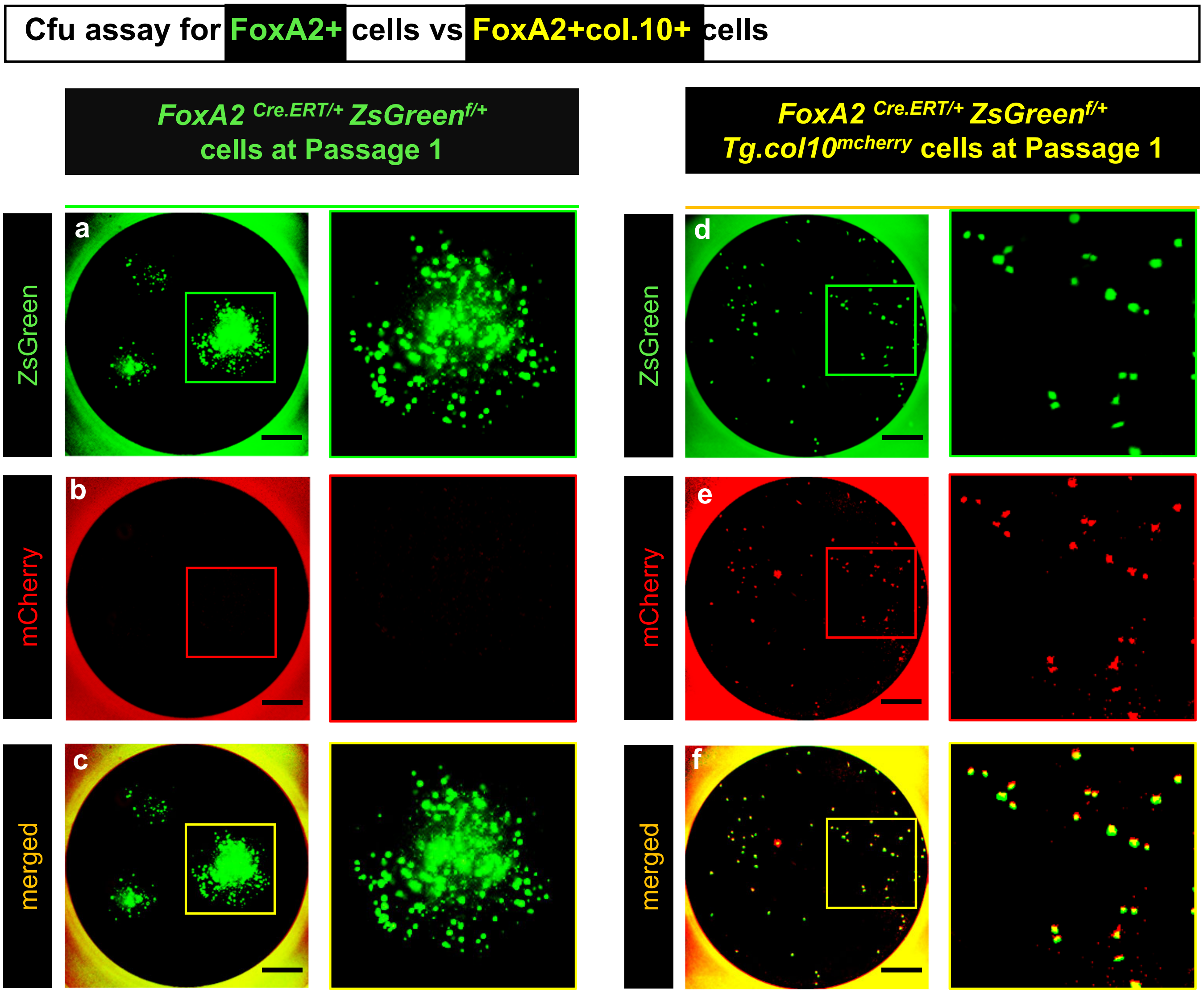

<u>Supplementary Fig.3</u> RZ FoxA2+ (green) cells form distinct primary colonies, whereas HZ FoxA2+col.10+ (yellow) cells fail to form colonies. Colony forming unit (cfu) assay from RZ FoxA2+ (ZsGreen+) cells (a-c) and HZ FoxA2+col.10+ (yellow) cells (d-f), isolated from *FoxA2*$^{CreERT2/+}$*; ZsGreen*$^{fl/+}$*; col.10*^{mcherry} mice treated with tamoxifen P13-P17 and harvested at P18. Fluorescence microscopy for ZsGreen (green), mcherry (red), merged (yellow). Insets, magnified view of a single colony, or individual cells. Scale bars, 1mm.

A. Colony size/shape

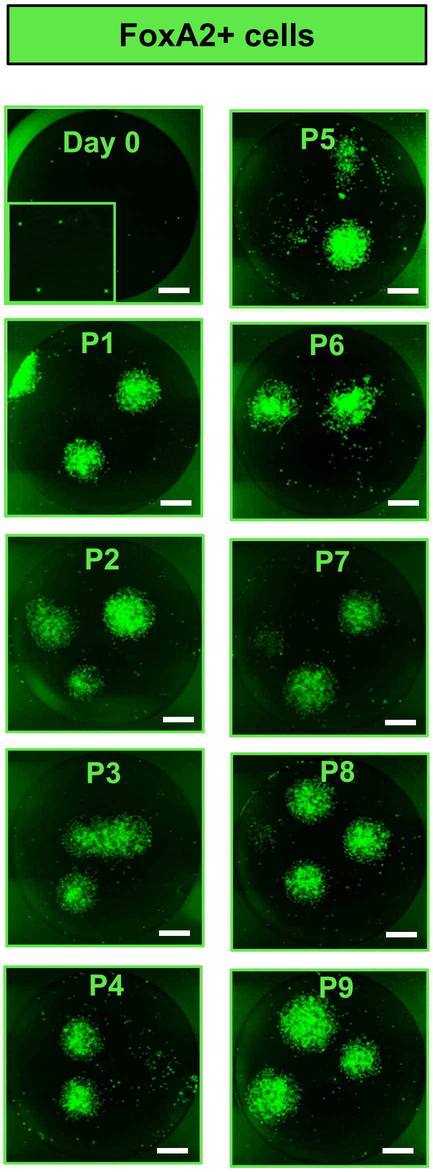

B. Number of colonies per passage

Passage #	FoxA2+ cells			PTHrP+ cells		
P1	50	29	33	50	46	57
P2	16	11	14	6	7	5
P3	9	4	11	2	3	1
P4	3	3	9	1	1	1
P5	2	2	8	1	1	
P6	2	2	8			
P7	1	2	7			
P8	1	2	7			
P9	1	2	7			

<u>Supplementary Fig.4</u> **FoxA2+ stem cells display more robust self-renewability than PTHrP+ stem cells. (A)** Colony forming unit (cfu) assay and subsequent passaging (P1-P9) of individual FoxA2+ (green) colonies. RZ FoxA2+ (green) cells were isolated from *FoxA2*$^{CreERT2/+}$*;ZsGreen*$^{fl/+}$*; col.10*^{mcherry} mice treated with tamoxifen P13-P17, and harvested at P18. Cells were seeded at clonal density in a 96 well plate. Scale bars, 1mm. **(B)** Comparison between the number of colonies established from FoxA2+cells versus PTHrP+cells. FoxA2+cells were isolated from *FoxA2*$^{CreERT2/+}$*; ZsGreen*$^{fl/+}$*;col.10*^{mcherry} mice, injected with tamoxifen P13 to P17, and harvested at P18. PTHrP+cells were isolated from P18 PTHrP^{mcherry} mice. Data at P1 (passage 1) reflects the number of colonies established from 10^3 cells per 10 cm petri dish. Data from passage P1 to P9 reflects the number of colonies that can be serially propagated, n=3 independent experiments for each cell type.

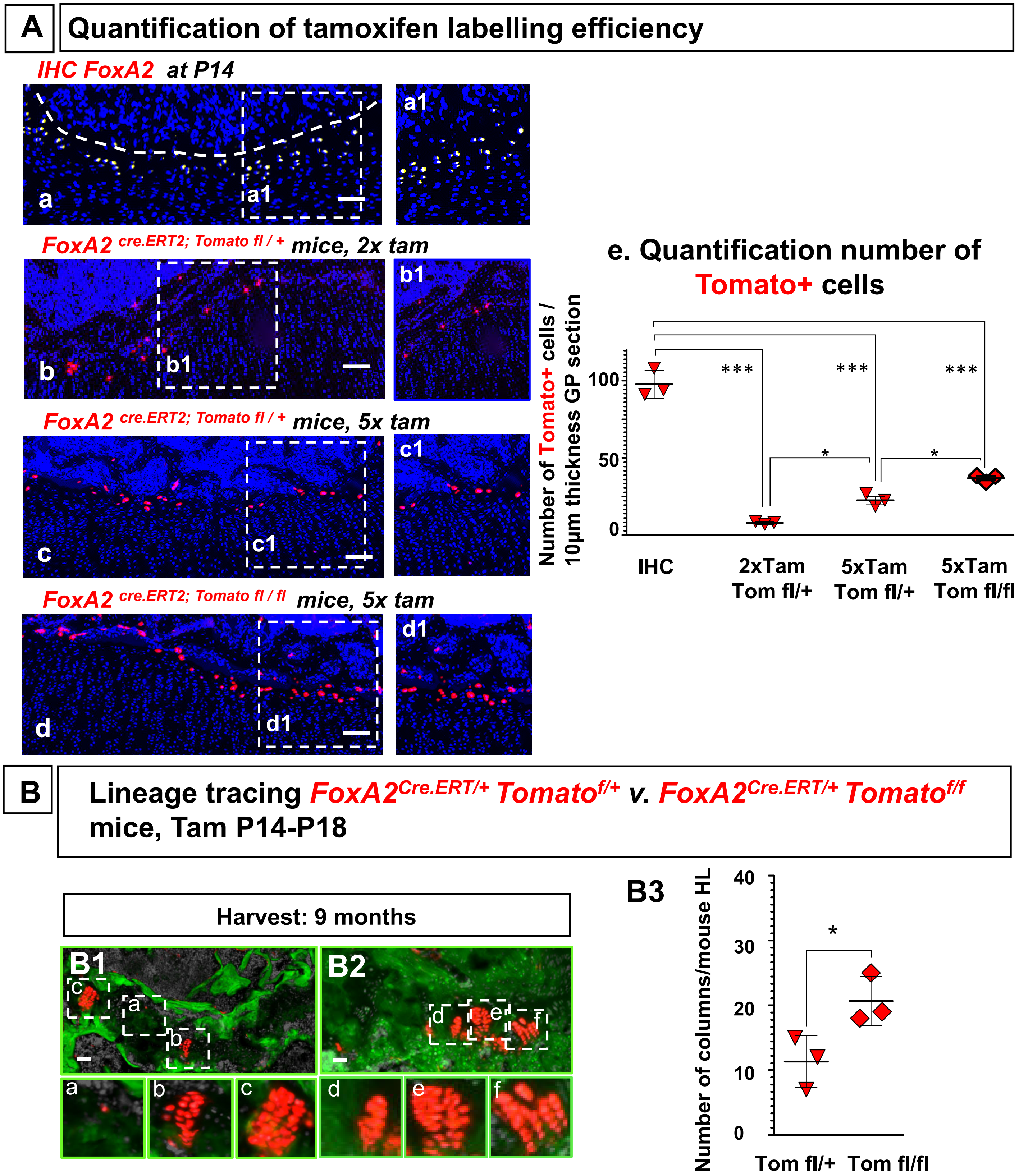

Supplementary Fig.5 Quantification tamoxifen labeling efficiency. **(A)** Tyramide amplification immunohistochemistry (IHC) for FoxA2 in P14 mice tibia sections (a). Tomato fluorescence microscopy in tibia sections from *FoxA2*$^{CreERT2/+}$*; Tomato*$^{fl/+}$ mice with 2x tamoxifen injections (P14-P15, harvest at P19) (b) *FoxA2*$^{CreERT2/+}$*;Tomato*$^{fl/+}$ mice with 5x tamoxifen injections (P14-P18, harvest at P19) (c), *FoxA2*$^{CreERT2/+}$*;Tomato*$^{fl/fl}$ mice with 5x tamoxifen injections (P14-P18, harvest at P19) (d). Scale bars, 50µm. Quantification of GP FoxA2+cells detected via IHC or by Tomato+ fluorescence per 10µm thickness GP section (e). Data are presented as mean±SD, n=3 mice. The asterisks indicate significant difference: One-way ANOVA, Tukey test; ***p = 8.6701E-08 (IHC vs 2xTam Tom fl/+), 4.43086E-07 (IHC vs 5xTam Tom fl/+), 2.43138E-06 (IHC vs 5xTam Tom fl/fl), *p = 0.033095466 (2xTam Tom fl/+ vs 5xTam Tom fl/+), 0.03696719 (5xTam Tom fl/+ vs 5xTam Tom fl/fl). Cont. next page

Supplementary Fig.5 Quantification tamoxifen labeling efficiency. **Cont.** **(B)** Tibia sections harvested from *FoxA2*$^{CreERT2/+}$; *Tomato*$^{fl/+}$ mice (B1) versus *FoxA2*$^{CreERT2/+}$;*Tomato*$^{fl/fl}$ mice (B2) treated with 5x tamoxifen injections (P14-P18), and harvested 9 months after the last injection. Tomato fluorescence (red), 5-DTAF (green), Hoechst dye (grey). Scale bars, 200µm. Representative details, from the section pictured above, shown in numbered insets (a-f). Quantification total number of Tomato+ columns of progeny, per mouse hindlimb (B3). Data are presented as mean±SD, n=3 mice. The asterisks indicate significant difference: Two-tailed Student's unpaired-samples t test; *p = 0.043278 (Tom fl/+ vs Tom fl/fl). Complete statistical information is provided in Supplementary Table 1.

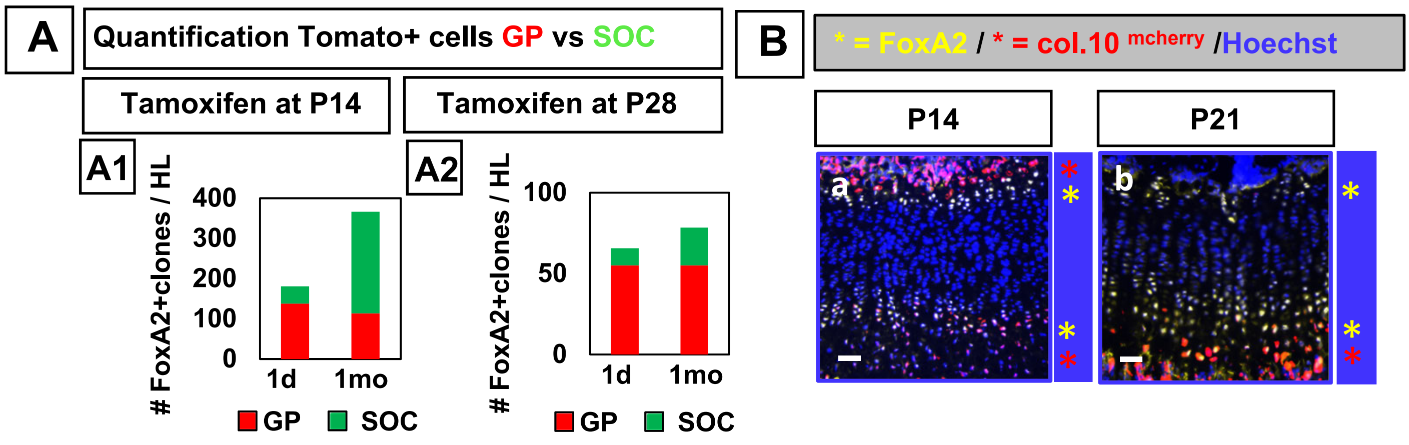

C Quantification total number of Tomato+ single cells, clusters, columns

FOXA2CRE/+ TOM/+ MICE TREATED WITH 5X TAMOXIFEN INJECTIONS STARTING AT P14, AND HARVESTED AT 1-DAY, 1-MO., 3-MO., AND 9-MO.

	1 day				1 mo.				3 mo.				9 mo.			
	SINGLE CELLS	CLUSTER	COLUMN	TOTAL CLONES	SINGLE CELLS	CLUSTER	COLUMN	TOTAL CLONES	SINGLE CELLS	CLUSTER	COLUMN	TOTAL CLONES	SINGLE CELLS	CLUSTER	COLUMN	TOTAL CLONES
MOUSE 1	106	35	0	141	101	72	1	174	31	16	4	51	13	9	15	37
MOUSE 2	103	14	0	117	48	42	1	91	24	13	1	38	32	16	12	60
MOUSE 3	128	27	0	155	41	33	1	75	18	13	4	35	10	14	7	31
Total Clones From All 3 Mice =				413				340				124				128

FOXA2CRE/+ TOM/+ MICE TREATED WITH 10X TAMOXIFEN INJECTIONS STARTING AT P28, AND HARVESTED AT 1-DAY, 1-MO., 3-MO., AND 9-MO.

	1 day				1 mo.				3 mo.				9 mo.			
	SINGLE CELLS	CLUSTER	COLUMN	TOTAL CLONES	SINGLE CELLS	CLUSTER	COLUMN	TOTAL CLONES	SINGLE CELLS	CLUSTER	COLUMN	TOTAL CLONES	SINGLE CELLS	CLUSTER	COLUMN	TOTAL CLONES
MOUSE 1	53	10	0	63	36	26	5	67	22	14	10	46	30	7	29	66
MOUSE 2	47	13	0	60	30	37	0	67	25	24	6	55	17	10	32	59
MOUSE 3	30	12	0	42	18	33	1	52	36	27	8	71	20	15	18	53
Total Clones From All 3 Mice =				165				186				172				178

Supplementary Fig.6 The dynamics of FoxA2+cells clonality. (A) Total number of Tomato+ cells quantified in growth plate (GP) and secondary ossification center (SOC), for FoxA2$^{CreERT2/+}$; Tomato$^{fl/+}$ mice treated with tamoxifen P14-P18, and harvested at 1-day and 1-month (A1), or treated with tamoxifen P28-P37, and harvested at 1-day and 1-month (A2). To quantify the number of FoxA2+ (Tomato+) cells present in the GP, we counted the Tomato+ cells in a domain extending 100μm away from the GP/SOC interface, towards the GP. To quantify the number of FoxA2+ (Tomato+) cells present in the SOC, we counted the Tomato+ cells in a domain extending 100μm away from the GP/SOC interface, towards the SOC. The number of Tomato+cells represents a sum of n= 8 sections per mouse hindlimb. **(B)** Immunohistochemistry for FoxA2, and fluorescence microscopy of Tg.col10^{mcherry} mice on P14 (a) and P21 (b) tibia sections. FoxA2 (yellow), mcherry fluorescence (red), Hoechst (blue). Scale bars, 50μm. **(C)** Quantification total number of Tomato+ clones (single cells, clusters, columns) from *FoxA2$^{CreERT2/+}$;Tomato$^{fl/+}$* mice injected with tamoxifen starting P14, or P28, and harvested 1-day, 1-, 3-, or 9- months after the last injection.

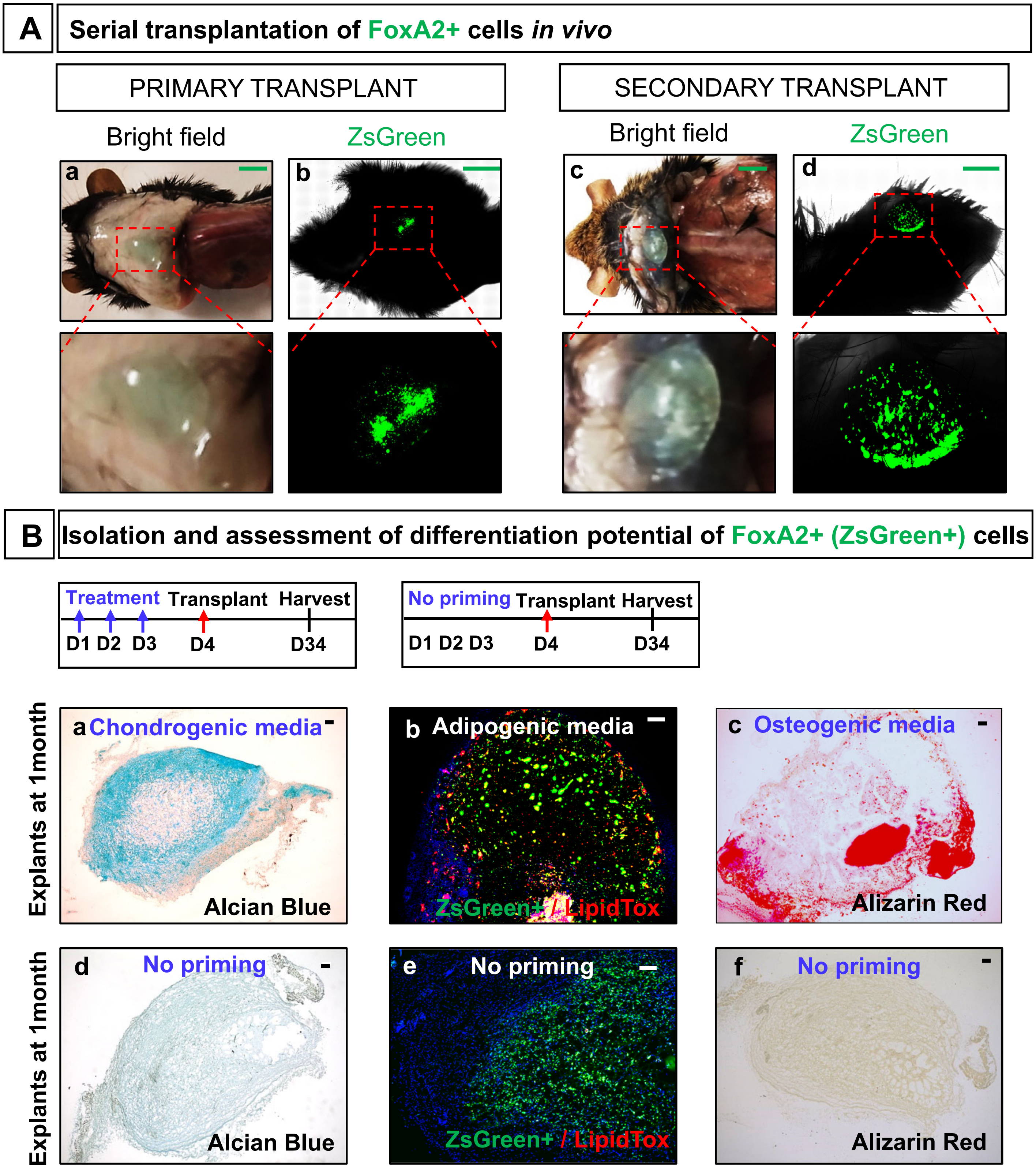

<u>Supplementary Fig.7</u> Isolation and assessment of differentiation potential of FoxA2+ (ZsGreen+) cells in the serial reconstitution assay. (A) Bright-field and fluorescence images showing the primary (a, b) and secondary (c, d) grafts 1-month after transplantation in secondary and tertiary recipient mice. Scale bars, 1 cm. **(B)** Alcian blue (a), LipidTox (b) or Alizarin Red-S (c) staining of cross-sections of grafts generated 30 days after transplantation of lineage-primed FoxA2+ cells under chondrogenic, adipogenic or osteogenic conditions. Alcian blue (d), LipidTox (e) or Alizarin Red-S (f) staining of cross-sections of grafts generated 30 days after transplantation of FoxA2+ cells, in the absence of lineage priming. Scale bar, 200 µm.

A SH1 – like GP injury measurements

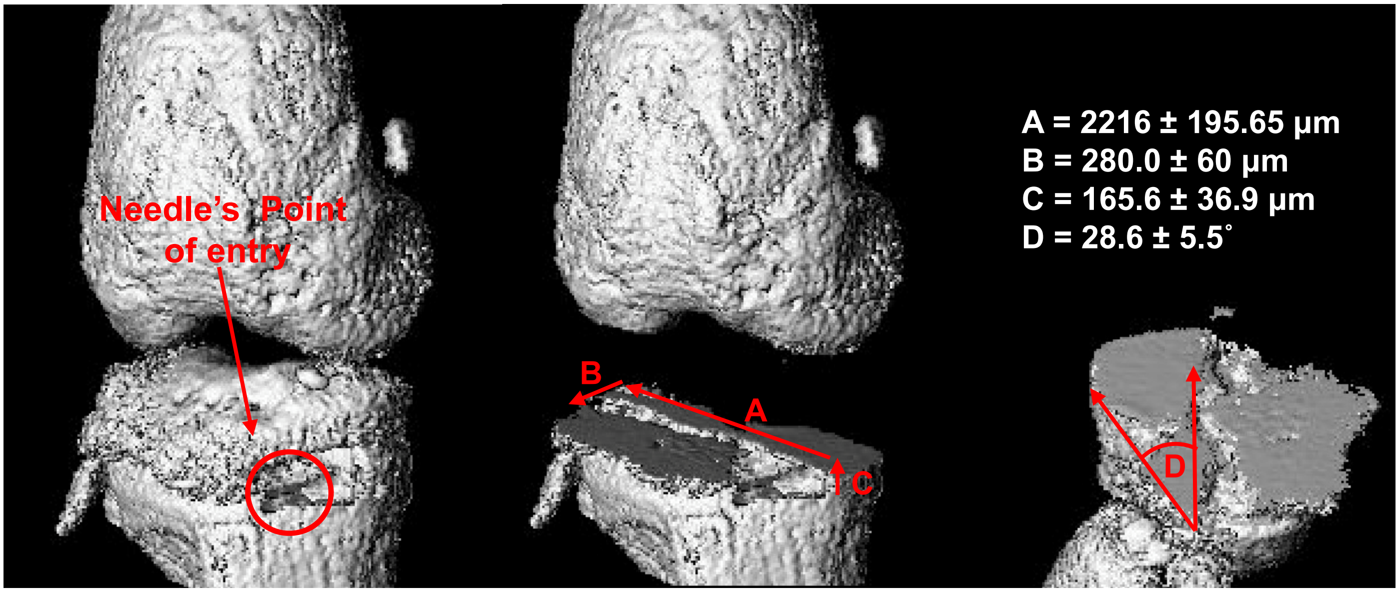

B Dimensions of the GP lesion

Mouse ID	A (µm)	B (µm)	C (µm)	D (Degree)
Mouse 1	2120	230	198	22.33
Mouse 2	2020	220	162	28.15
Mouse 3	2200	370	162	31.39
Mouse 4	2540	290	108	24.81
Mouse 5	2200	290	198	36.2

C Tibia growth after SH1 – like GP injury

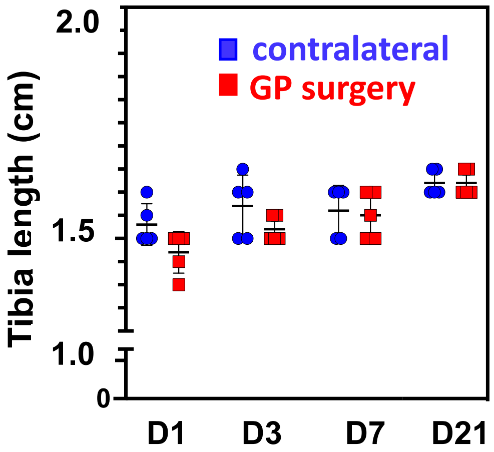

<u>Supplementary Fig.8</u> **Dimensions of the GP lesion after SH1–like surgery. (A)** A 3D reconstruction of the injured growth plate (GP) proximal tibia, 1-day after surgery, exposure to CA4+. Visualization of the defect, induced by the SH1 (Salter Harris type 1)-like surgery, in the tibial physis. Average dimensions, and angle relative to the coronal plane, from n=5 independent surgeries. CECT measurements of the SH1-like injury: A (lateral to medial), B (anterior to posterior), C (height), D (angle). **(B)** Dimensions of the GP lesion, n=5 surgeries. **(C)** Quantification of the total tibial length at 1-day, 3-days, 7-days, and 21-days after SH1-like surgery in both the operated leg and the contralateral leg. Data are presented as mean±SD, n=5 mice. Complete statistical information is provided in Supplementary Table 1.

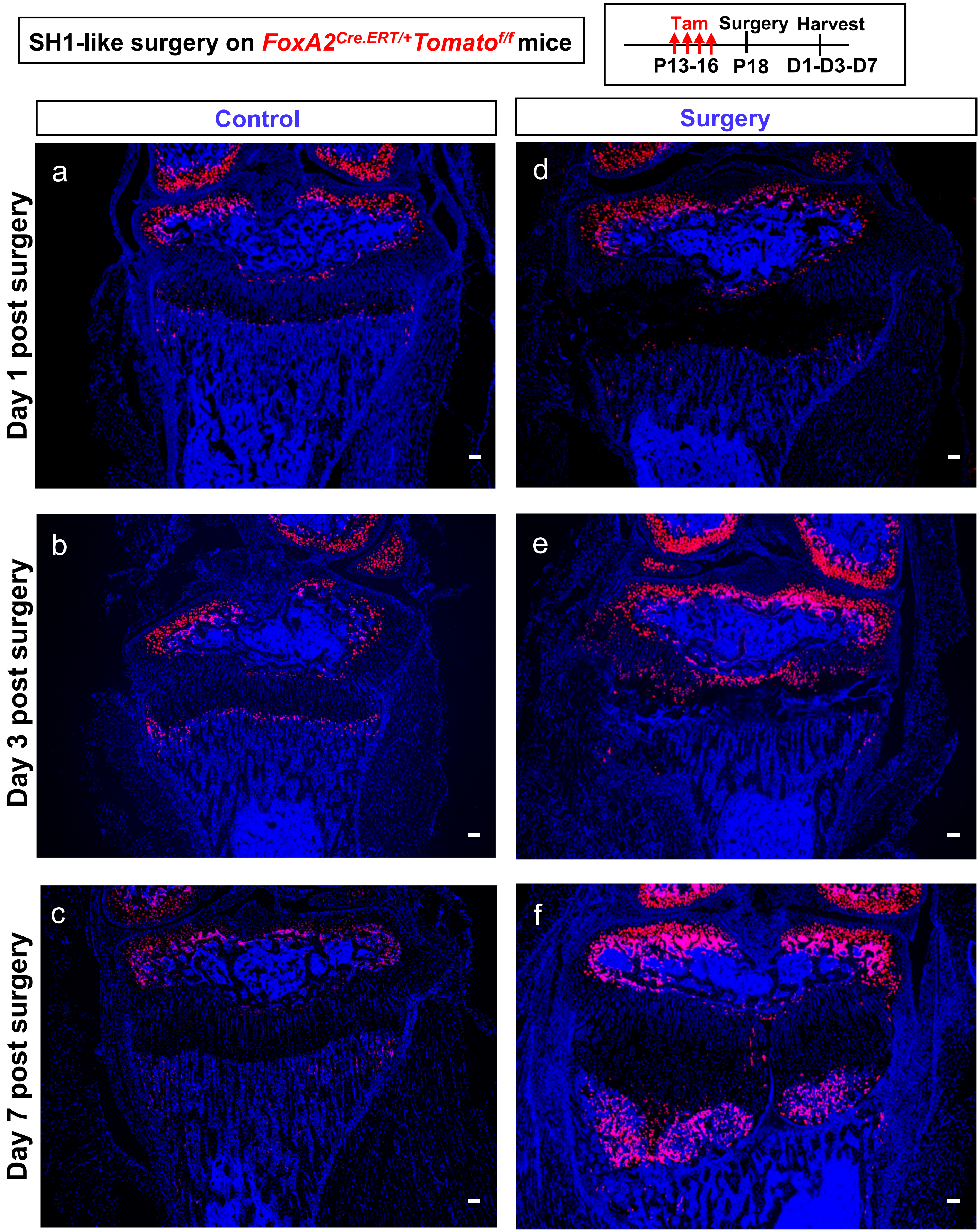

<u>Supplementary Fig.9</u> FoxA2+cells expand in response to SH1-like surgery. Control (a-c) and operated (d-f) tibia from FoxA2$^{CreERT2/+}$;Tomato$^{fl/fl}$ mice, injected with tamoxifen (Tam) P13-P16, operated via SH1 (Salter Harris type 1)-like surgery at P18, and harvested at 1-, 3-, 7-days post-op. Tomato fluorescence (red), Hoechst (blue), 4x magnification, whole tibia sections. Scale bars, 100µm.

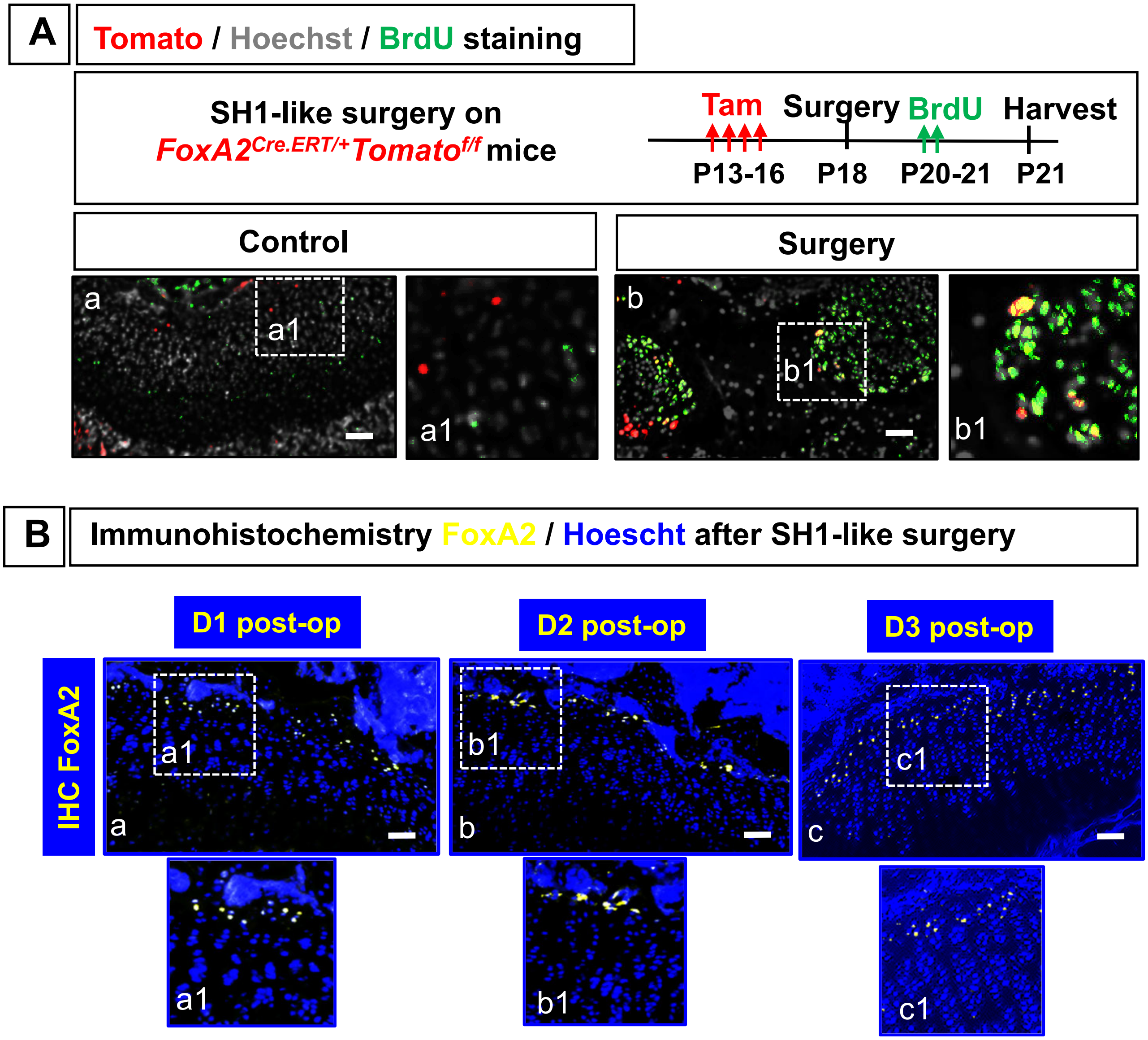

<u>Supplementary Fig.10</u> FoxA2+cells are BrdU positive. (A) Control (a) and operated (b) tibia from FoxA2$^{CreERT2/+}$;Tomato$^{fl/fl}$ mice, injected with tamoxifen (Tam) P13-P16, operated at P18 via SH1 (Salter Harris type 1)-like surgery, treated with 3x BrdU injections administrated P20-P21, harvested at P21 (3-days post-op). Tomato fluorescence (red), Hoechst (blue), BrdU (green). Insets, magnified view of injury (b1) or control (a1). Scale bars, 100μm. **(B)** Immunohistochemistry (IHC) for FoxA2, on operated tibias harvested at 1-day (a), 2-days (b), 3-days (c) after SH1-like surgery. FoxA2 (yellow), Hoechst (blue). Insets, magnified view of injury (a1-c1). Scale bars, 100μm.

Gating Strategy

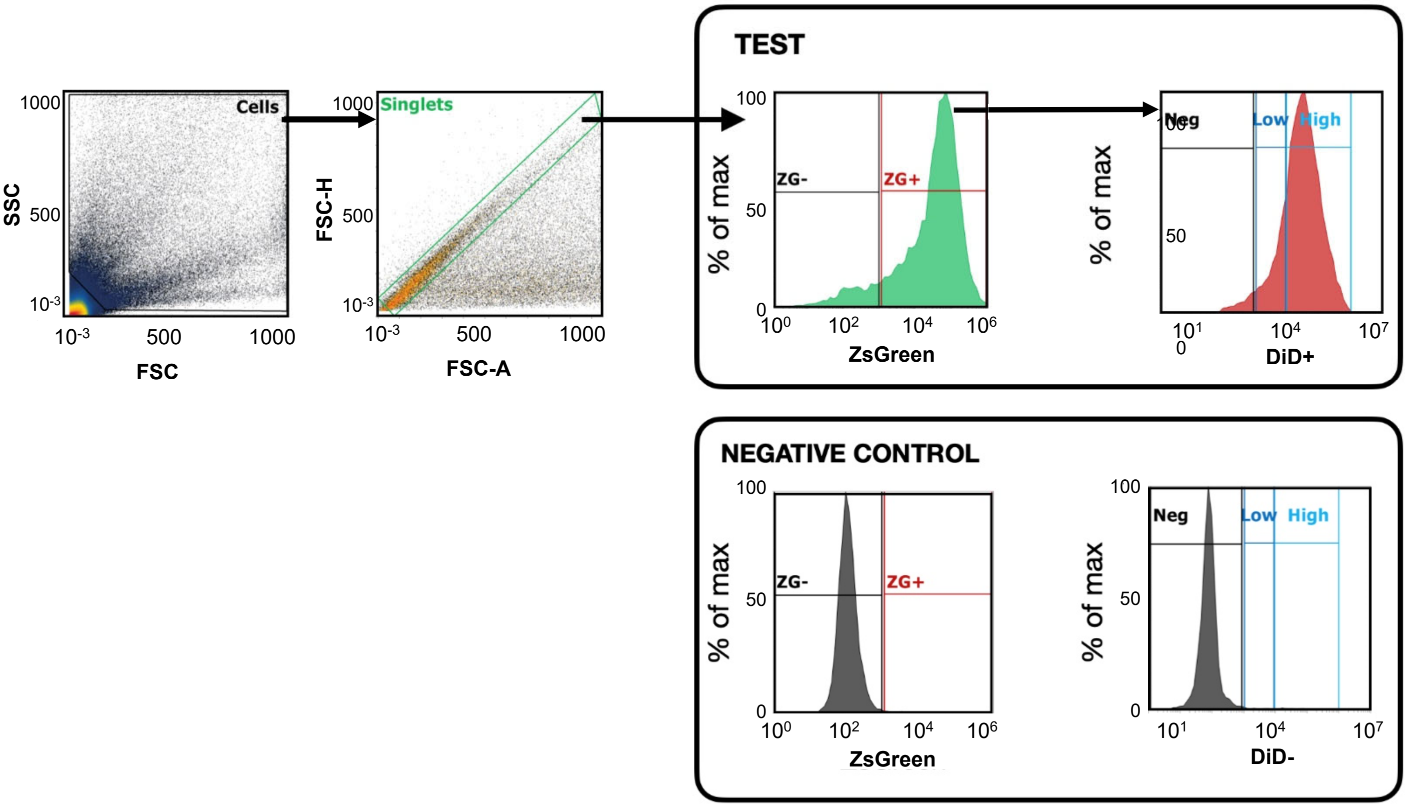

<u>Supplementary Fig.11</u> **Gating strategy used for proliferation tracking to test self-renewal property of transplanted FoxA2+ (ZsGreen+) cells in the serial *in vivo* reconstitution assay.** Representative FACS plots showing gating scheme for defining DiD^{high} and DiD^{low} subpopulations within the ZsGreen+ population of cells isolated from the primary and secondary FoxA2+ cell grafts. After exclusion of doublets, ZsGreen+ cells were gated and the proportion of DiD^{high} and DiD^{low} cells was evaluated.

Supplementary Table S1

Figure	Dataset	Statistical test	Multiple comparison test	n	Exact P value
**Fig. 2A**	P3-4 GP vs P3-4 SOC	One-way ANOVA	Tukey	3	0.000003
	P7-8 GP vs P7-8 SOC	One-way ANOVA	Tukey	3	0.000005
	P13-14 GP vs P13-14 SOC	One-way ANOVA	Tukey	3	0.980399
	P13-17 GP vs P13-17 SOC	One-way ANOVA	Tukey	3	0.011031
	P3-4 GP vs P13-17 GP	One-way ANOVA	Tukey	3	0.000569
	P7-8 GP vs P13-17 GP	One-way ANOVA	Tukey	3	0.000828
**Fig. 5B (d)**	D0 vs D30	unpaired two-tailed t test	_	3	7.0148E-07
**Fig. 5B (e)**	D0 vs D30	unpaired two-tailed t test	_	3	7.84512E-06
**Fig. 5B (g)**	D0 vs D30	unpaired two-tailed t test	_	3	3.32176E-06
**Fig. 5B (h)**	D0 vs D30	unpaired two-tailed t test	_	3	3.46695E-06
**Fig. 6D**	d1 vs d3	unpaired two-tailed t test	_	5	0.055556
	d1 vs d7	unpaired two-tailed t test	_	5	0.0079
**Fig. 6F**	d1 vs d3	unpaired two-tailed t test	_	3	0.037861
	d3 vs d7	unpaired two-tailed t test	_	3	0.007121
	d1 vs d7	unpaired two-tailed t test	_	3	0.000839
**Fig. 7B b1. GP**	Day1: control vs operated	One-way ANOVA	Tukey	4	0.391815
	Day3: control vs operated	One-way ANOVA	Tukey	4	0.032625
	Day7: control vs operated	One-way ANOVA	Tukey	4	0.999984
**Fig. 7B b2. SOC**	Day1: control vs operated	One-way ANOVA	Tukey	4	0.999069
	Day3: control vs operated	One-way ANOVA	Tukey	4	0.998693
	Day7: control vs operated	One-way ANOVA	Tukey	4	0.995554
**Fig. 7B b3. Metaphysis**	Day1: control vs operated	One-way ANOVA	Tukey	4	0.036130
	Day3: control vs operated	One-way ANOVA	Tukey	4	0.021045
	Day7: control vs operated	One-way ANOVA	Tukey	4	0.0000003
**Fig. 8B**	CreTom vs CreTom DTA	unpaired two-tailed t test	_	4	0.028571
**Fig. 8D**	DTA/DTA vs CreDTA/DTA	unpaired two-tailed t test	_	5	0.007937
**Supplementary Fig. 1A**	GP vs SOC	unpaired two-tailed t test	_	4	0.000082
**Supplementary Fig. 2B**	**Aggrecan:**				
	FoxA2+ cells vs PTHRP+ cells	One-way ANOVA	Tukey	4	0.436345154
	FoxA2+ cells vs BMSCs	One-way ANOVA	Tukey	4	2.61879E-08
	PTHRP+ cells vs BMSCs	One-way ANOVA	Tukey	4	3.09872E-05
	**COL2A1:**				
	FoxA2+ cells vs PTHRP+ cells	One-way ANOVA	Tukey	4	0.970384408
	FoxA2+ cells vs BMSCs	One-way ANOVA	Tukey	4	1.1691E-07
	PTHRP+ cells vs BMSCs	One-way ANOVA	Tukey	4	5.89962E-06
	**COL1A1:**				
	FoxA2+ cells vs PTHRP+ cells	One-way ANOVA	Tukey	4	>0.99999999
	FoxA2+ cells vs BMSCs	One-way ANOVA	Tukey	4	2.65003E-07
	PTHRP+ cells vs BMSCs	One-way ANOVA	Tukey	4	2.51267E-07
	**ALP:**				
	FoxA2+ cells vs PTHRP+ cells	One-way ANOVA	Tukey	4	>0.99999999
	FoxA2+ cells vs BMSCs	One-way ANOVA	Tukey	4	7.20261E-05
	PTHRP+ cells vs BMSCs	One-way ANOVA	Tukey	4	6.00535E-05
**Supplementary Fig. 5A**	IHC vs. 2xT	One-way ANOVA	Tukey	3	8.6701E-08
	IHC vs. 5xT	One-way ANOVA	Tukey	3	4.43086E-07
	IHC vs. 5xTT	One-way ANOVA	Tukey	3	2.43138E-06
	2xT vs. 5xT	One-way ANOVA	Tukey	3	0.033095466
	5xT vs. 5xTT	One-way ANOVA	Tukey	3	0.03696719
**Supplementary Fig. 5B**	Tom/+ vs Tom/Tom	unpaired two-tailed t test	_	3	0.043278
**Supplementary Fig. 8C**	Day1: control vs operated	One-way ANOVA	Tukey	5	0.433288
	Day3: control vs operated	One-way ANOVA	Tukey	5	0.653546
	Day7: control vs operated	One-way ANOVA	Tukey	5	0.999961
	Day21: control vs operated	One-way ANOVA	Tukey	5	>0.999999

Supplementary Table 1 Statistical tests and p-values for quantitative data.
