# Supplementary material for: A FoxA2+ long-term stem cell population is necessary for growth plate cartilage regeneration after injury
Source: Nat Commun. 2022 May 6;13:2515. doi: 10.1038/s41467-022-30247-1 (PMC9076650; doi:10.1038/s41467-022-30247-1)
Supplement: Supplementary file 2 — Reporting Summary [file 41467_2022_30247_MOESM2_ESM.pdf]

nature research

Corresponding author(s): Andreia Ionescu

Last updated by author(s): 11th April 2022

Reporting Summary

Nature Research wishes to improve the reproducibility of the work that we publish. This form provides structure for consistency and transparency in reporting. For further information on Nature Research policies, seeAuthors & Referees and theEditorial Policy Checklist .

Statistics

For all statistical analyses, confirm that the following items are present in the figure legend, table legend, main text, or Methods section.

n/a	Confirmed	
☐	☒	The exact sample size (*n*) for each experimental group/condition, given as a discrete number and unit of measurement
☐	☒	A statement on whether measurements were taken from distinct samples or whether the same sample was measured repeatedly
☐	☒	The statistical test(s) used AND whether they are one- or two-sided<br>*Only common tests should be described solely by name; describe more complex techniques in the Methods section.*
☐	☒	A description of all covariates tested
☐	☒	A description of any assumptions or corrections, such as tests of normality and adjustment for multiple comparisons
☐	☒	A full description of the statistical parameters including central tendency (e.g. means) or other basic estimates (e.g. regression coefficient) AND variation (e.g. standard deviation) or associated estimates of uncertainty (e.g. confidence intervals)
☐	☒	For null hypothesis testing, the test statistic (e.g. *F*, *t*, *r*) with confidence intervals, effect sizes, degrees of freedom and *P* value noted<br>*Give P values as exact values whenever suitable.*
☒	☐	For Bayesian analysis, information on the choice of priors and Markov chain Monte Carlo settings
☒	☐	For hierarchical and complex designs, identification of the appropriate level for tests and full reporting of outcomes
☒	☐	Estimates of effect sizes (e.g. Cohen's *d*, Pearson's *r*), indicating how they were calculated

Our web collection on statistics for biologists contains articles on many of the points above.

Software and code

Policy information about availability of computer code

Data collection	Keyence BZ-X710 All-in-One Fluorescence Microscope SYSTEM, FACSAria II (BD)
Data analysis	Image J (NIH), FCS Express, GraphPad Prism 5.0

For manuscripts utilizing custom algorithms or software that are central to the research but not yet described in published literature, software must be made available to editors/reviewers. We strongly encourage code deposition in a community repository (e.g. GitHub). See the Nature Research guidelines for submitting code & software for further information.

Data

Policy information about availability of data

All manuscripts must include a data availability statement. This statement should provide the following information, where applicable:

- Accession codes, unique identifiers, or web links for publicly available datasets
- A list of figures that have associated raw data
- A description of any restrictions on data availability

The data that support the findings of this study are available from the corresponding author upon reasonable request.

Field-specific reporting

Please select the one below that is the best fit for your research. If you are not sure, read the appropriate sections before making your selection.

☒ Life sciences ☐ Behavioural & social sciences ☐ Ecological, evolutionary & environmental sciences

For a reference copy of the document with all sections, see nature.com/documents/nr-reporting-summary-flat.pdf

Life sciences study design

All studies must disclose on these points even when the disclosure is negative.

Sample size	Group size was calculated using the application available in http://www.biomath.info/power/ttest.htm based upon α = 0.05 and power of β = 0.8
Data exclusions	No animals or samples were excluded from the analyses.
Replication	For all data presented in the manuscript, we examined at least three independent biological samples to ensure reproducibility. For each series of experiments, all attempts at replication were successful.
Randomization	Randomization methods were not employed because the Cre+ and Cre- groups were littermates that were housed together with the same mother and they were of same age, same sex and received the same treatments.
Blinding	Both the surgeon performing the GP surgery and the observer measuring the extent of GP damage or counting fluorescent cells were blinded to the experimental groups.

Reporting for specific materials, systems and methods

We require information from authors about some types of materials, experimental systems and methods used in many studies. Here, indicate whether each material, system or method listed is relevant to your study. If you are not sure if a list item applies to your research, read the appropriate section before selecting a response.

Materials & experimental systems

n/a	Involved in the study	
☐	☒	Antibodies
☒	☐	Eukaryotic cell lines
☒	☐	Palaeontology
☐	☒	Animals and other organisms
☒	☐	Human research participants
☒	☐	Clinical data

Methods

n/a	Involved in the study	
☒	☐	ChIP-seq
☐	☒	Flow cytometry
☒	☐	MRI-based neuroimaging

Antibodies

Antibodies used	FoxA1 (Abcam, Cat#ab23738, Lot#GR3276275-1), 1:500<br>FoxA2 (Millipore, Cat#07-633, Lot#2768454), 1:2000<br>FoxA3 (Santa-Cruz, 375 Cat#sc-25357, Lot#A1904), 1:50<br>Ki67 (Cell Signaling, Cat#9129S, Lot#3), 1:800<br>BrdU 376 (ThermoFisher, Cat# B35138, Lot#1774716), 1:100
Validation	More detailed information about these antibodies is available on the manufacturer's website.

Animals and other organisms

Policy information about studies involving animals; ARRIVE guidelines recommended for reporting animal research

Laboratory animals	Laboratory mice (Mus musculus) were the model species used for this study. We used transgenic mouse models and genetically engineered mouse models utilizing the Cre-Lox recombination system. The genetically modified mouse lines used in this study were backcrossed to a C57Bl/6J background. Mice with both sexes (males and females, randomized in groups) were used up to 1 year of age. Mouse strains used in this study were as follows: FoxA2CreERT2/+ mice (JAX stock#008464), Tomatofl/fl mice (JAX stock#007914), ZsGreenfl/fl mice (JAX stock #007906), DTAf/f mice (JAX stock#009669), AggCreERT2/+ mice (JAX stock 019148), Tg.col1a1*2.3-gfp mice (JAX stock 013134), PTHrPmcherry mice (JAX stock#032872) and Tg.col10a1-mCherry mice (JAX stock#017465).
Wild animals	n/a
Field-collected samples	n/a
Ethics oversight	All procedures involving mice were performed with approved protocols from Northeastern University Institutional Animal Care and Use Committee (IACUC), protocol#20-0625R (Ionescu).

Note that full information on the approval of the study protocol must also be provided in the manuscript.

Flow Cytometry

Plots

Confirm that:

- [x] The axis labels state the marker and fluorochrome used (e.g. CD4-FITC).
- [x] The axis scales are clearly visible. Include numbers along axes only for bottom left plot of group (a 'group' is an analysis of identical markers).
- [x] All plots are contour plots with outliers or pseudocolor plots.
- [x] A numerical value for number of cells or percentage (with statistics) is provided.

Methodology

Sample preparation	GP cartilage was dissected out of proximal tibia, distal radius and distal ulna of FoxA2CreERT2/+;ZsGreen fl/+;Tg.col10a1-mCherry mice or PTHrPmcherry mice. The surrounding tissues adhering to the growth plate were removed and the GP cartilage was subjected to predigestion with 0.2% Collagenase P (Cat# 11213857001, Sigma-Aldrich, USA) in DMEM high glucose (Cat# 11885084, Life Technologies, ThermoFisher, USA) in a shaking waterbath at 37C, 150 rpm for 40 minutes. The predigested growth plates were cut into 1-2 mm pieces with a sterile scalpel and subjected to final digestion with enzymes, 0.1 % collagenase II (Cat# 17101-015, Life Technologies, ThermoFisher, USA) and 0.05 % trypsin (Cat# 25200- 072, ThermoFisher, USA) at 37C in a CO2 incubator for 4 h. After complete digestion, the cell suspension was passed through a 70 um cell strainer, centrifuged at 1000xg for 20 minutes and the pellet was resuspended in complete growth medium (DMEM containing high glucose (4.5 g/liter), 1% L-glutamine, 10% fetal bovine serum, 100 U/ml of penicillin, and 100 mg/ml of streptomycin). For Fluorescence activated cell sorting (FACS), on average 500,000 cells (from 5-6 mice) were washed 2x with 2% heat-inactivated fetal calf serum in PBS, and then re-suspended in PBS. The analysis and sorting for ZsGreen or mcherry were performed with BD FACS Aria II equipped with six-laser system 355, 405, 445, 488, 561, and 641 nm. All FACS images show the representative contour plots derived from the analysis of three independent litters.
Instrument	FACSAria II (BD Biosciences)
Software	FCS Express Flow Cytometry software
Cell population abundance	n/a
Gating strategy	Single cells were first gated using FSC and SSC denominators. Samples derived from wild-type control mice were always used as a reference to determine the demarcation between the positive and negative populations.

- [x] Tick this box to confirm that a figure exemplifying the gating strategy is provided in the Supplementary Information.
